# BMP2 and BMP7 cooperate with H3.3K27M to promote quiescence and invasiveness in pediatric diffuse midline gliomas

**DOI:** 10.7554/eLife.91313

**Published:** 2024-10-07

**Authors:** Paul Huchede, Swann Meyer, Clément Berthelot, Maud Hamadou, Adrien Bertrand-Chapel, Andria Rakotomalala, Line Manceau, Julia Tomine, Nicolas Lespinasse, Paul Lewandowski, Martine Cordier-Bussat, Laura Broutier, Aurélie Dutour, Isabelle Rochet, Jean-Yves Blay, Cyril Degletagne, Valéry Attignon, Angel Montero-Carcaboso, Marion Le Grand, Eddy Pasquier, Alexandre Vasiljevic, Pascale Gilardi-Hebenstreit, Samuel Meignan, Pierre Leblond, Vanessa Ribes, Erika Cosset, Marie Castets

**Affiliations:** 1 Childhood Cancer & Cell Death (C3) team, LabEx DEVweCAN, Institut Convergence Plascan, Centre Léon Bérard, Centre de Recherche en Cancérologie de Lyon (CRCL), Université Claude Bernard Lyon 1, INSERM 1052, CNRS 5286 Lyon France; 2 University of Lille, CNRS, Inserm, CHU Lille, UMR9020-U1277-CANTHER Cancer Heterogeneity Plasticity and Resistance to Therapies, Centre Oscar Lambret Lille France; 3 Université Paris Cité, CNRS, Institut Jacques Monod Paris France; 4 Multisite Institute of Pathology, Groupement Hospitalier Est du CHU de Lyon, Hôpital Femme-Mère Enfant Bron France; 5 Platform of Cancer Genomics, Centre Léon Bérard Lyon France; 6 Preclinical Therapeutics and Drug Delivery Research Program, Department of Oncology, Hospital Sant Joan de Déu Barcelona Spain; 7 Centre de Recherche en Cancérologie de Marseille (CRCM), Université Aix-Marseille, Institut Paoli- Calmettes, Centre de Lutte Contre le Cancer de la région PACA, INSERM 1068, CNRS 7258 Marseille France; 8 Department of Pediatric Oncology, Institute of Pediatric Hematology and Oncology (IHOPe), Centre Léon Bérard Lyon France; 9 GLIMMER Of lIght (GLIoblastoma MetabolisM, HetERogeneity, and OrganoIds) team, Centre Léon Bérard, Centre de Recherche en Cancérologie de Lyon (CRCL), Université Claude Bernard Lyon 1, INSERM 1052, CNRS 5286 Lyon France; Queens University Canada; Fox Chase Cancer Center United States

**Keywords:** BMP, H3K27 mutation, DMG, invasion, Human

## Abstract

Pediatric diffuse midline gliomas (pDMG) are an aggressive type of childhood cancer with a fatal outcome. Their major epigenetic determinism has become clear, notably with the identification of K27M mutations in histone H3. However, the synergistic oncogenic mechanisms that induce and maintain tumor cell phenotype have yet to be deciphered. In 20 to 30% of cases, these tumors have an altered BMP signaling pathway with an oncogenic mutation on the BMP type I receptor ALK2, encoded by *ACVR1*. However, the potential impact of the BMP pathway in tumors non-mutated for *ACVR1* is less clear. By integrating bulk, single-cell, and spatial transcriptomic data, we show here that the BMP signaling pathway is activated at similar levels between *ACVR1* wild-type and mutant tumors and identify BMP2 and BMP7 as putative activators of the pathway in a specific subpopulation of cells. By using both pediatric isogenic glioma lines genetically modified to overexpress H3.3K27M and patients-derived DIPG cell lines, we demonstrate that BMP2/7 synergizes with H3.3K27M to induce a transcriptomic rewiring associated with a quiescent but invasive cell state. These data suggest a generic oncogenic role for the BMP pathway in gliomagenesis of pDMG and pave the way for specific targeting of downstream effectors mediating the K27M/BMP crosstalk.

## Introduction

pDMG, including Diffuse Intrinsic Pontine Gliomas (DIPG), are rare and aggressive brain tumors that arise in the pons, thalamus, or spinal cord of children, most commonly between the ages of 5 and 10 (Mackay et al., 2017; Di Ruscio et al., 2022; Sulman and Eisenstat, 2021). pDMG are almost uniformly fatal, with a median overall survival of 9–11 months (Khuong-Quang et al., 2012; Hoffman et al., 2018), thereby representing the leading cause of mortality in pediatric neuro-oncology (Mackay et al., 2017; Sulman and Eisenstat, 2021). Clinical management of pDMG and especially of DIPG is a major challenge given their location in vital nervous centers and their leptomeningeal dissemination, which prevent any prospect of surgical intervention (Sethi et al., 2011). Radiotherapy, the current standard of care, is at best only transiently effective (Sulman and Eisenstat, 2021; Vitanza and Monje, 2019). Moreover, pDMG are highly resistant to currently available chemotherapies although promising combinations of molecules, including ONC201, are currently in clinical trials (Jackson et al., 2023).

High-throughput sequencing demonstrated that pDMG is associated with a major disruption of the epigenetic landscape, resulting in 80% of cases from a lysine-to-methionine substitution at position 27 (K27M) in genes encoding histone variants H3.3 (*H3F3A/H3-3A*) or H3.1 (*HIST1H3B/H3C2* or *HIST1H3C/H3C3*) (Khuong-Quang et al., 2012; Schwartzentruber et al., 2012; Sturm et al., 2012; Wu et al., 2012). H3K27M variants, also known as oncohistones, are affine for EZH2, the methyltransferase subunit of Polycomb Repressive Complex 2 (PRC2) and inhibit its activity. As a result, pDMG show abnormal methyl group deposition on H3K27 and a global loss of trimethylated H3K27 (H3K27me3) (Bender et al., 2013; Chan et al., 2013; Lewis et al., 2013; Piunti et al., 2017; Stafford et al., 2018; Harutyunyan et al., 2019), a histone mark associated with transcriptional repression.

In addition to *TP53*, other genetic alterations have been described, notably in the *PDGFR*, *EGFR,* or *PIK3CA* genes (Mackay et al., 2017), suggesting an oncogenic synergy between H3K27-based epigenetic remodeling and the activation of several transcriptional programs (Nagaraja et al., 2019; Huchedé et al., 2022). Accordingly, 20 to 30% of pDMG cases are associated with mutations in the *ACVR1* gene, encoding for the bone morphogenetic protein (BMP) type I receptor ALK2 (Buczkowicz et al., 2014; Fontebasso et al., 2014; Taylor et al., 2014; Wu et al., 2014), which leads to the overactivation of intracellular BMP signaling pathway (Carvalho et al., 2019; Fortin et al., 2020; Haupt et al., 2018; Hoeman et al., 2019; Ramachandran et al., 2021; Jessa et al., 2022; Mucha et al., 2018; Hatsell et al., 2015; Hino et al., 2015). Such increase in BMP signaling in the pDMG epigenetic context has been suggested to promote tumorigenesis by maintaining cells in a proliferative, mesenchymal-like, and undifferentiated state in vitro and in vivo *(*Carvalho et al., 2019; Fortin et al., 2020; Hoeman et al., 2019; Jessa et al., 2022). In 69% of cases, these *ACVR1* mutations are associated with the H3.1K27M mutation, whereas less than 20% are observed in H3.3K27M tumors (Mackay et al., 2017). The question is then whether the BMP signaling pathway could also be involved in the tumorigenesis of H3.3K27M-positive tumors. Recent data support the idea that H3.1K27M tumors mutated for *ACVR1* would emerge from a ventral pool of oligodendrocyte progenitor cells (OPC) characterized by the expression of the transcription factor NKX6-1 and dependent on the Sonic Hedgehog (SHH) signaling (Jessa et al., 2022). In contrast, H3.3K27M is thought to preferentially derive from dorsal progenitors expressing the dorsal PAX3- and BMP-dependent transcription factor (Jessa et al., 2022). In other words, H3.1K27M tumors would depend on the concomitant acquisition of the *ACVR1* mutation for transformation, whereas the BMP pathway could be activated in a tumor-independent manner in H3.3K27M tumors, due to their BMP-rich microenvironment. However, while the pro-tumorigenic activity of BMP signaling is clear in *ACVR1* mutant pDMG, recent data have unexpectedly reported that BMP ligands may exert a tumor-suppressive activity in H3.3K27M-*ACVR1* wild-type (WT) pDMG cellular models (Sun et al., 2022).

Here, we have integrated bulk, single-cell and spatial transcriptomic data from patients with functional approaches in cellular models to characterize the impact of BMP activation in H3.3K27M-*ACVR1* WT pDMG. Bioinformatic analyses indicate that BMP signaling pathway activation ground state is independent of *ACVR1* status in pDMG, which likely results both from BMP2/7 tumor-autonomous and microenvironment-driven signals in *ACVR1* WT tumors. Functional modeling on pediatric glioma cell lines and spatial transcriptomics unveil that H3.3K27M and BMP2/7 synergize to induce a transcriptomic switch leading to a quiescent but invasive cell state. These results shed new light on the complex phenotype resulting from the synergy between activation of the BMP pathway and epigenetic remodeling induced by the H3.3K27M mutation in pDMG, and the interest of a therapeutic approach targeting the downstream oncogenic pathways responsible for the invasive potential of tumor cells.

## Results

### BMP pathway activation level is independent of *ACVR1* mutational status in pDMG, and likely supported by BMP2/BMP7 in H3.3K27M tumors

**Figure 1.**
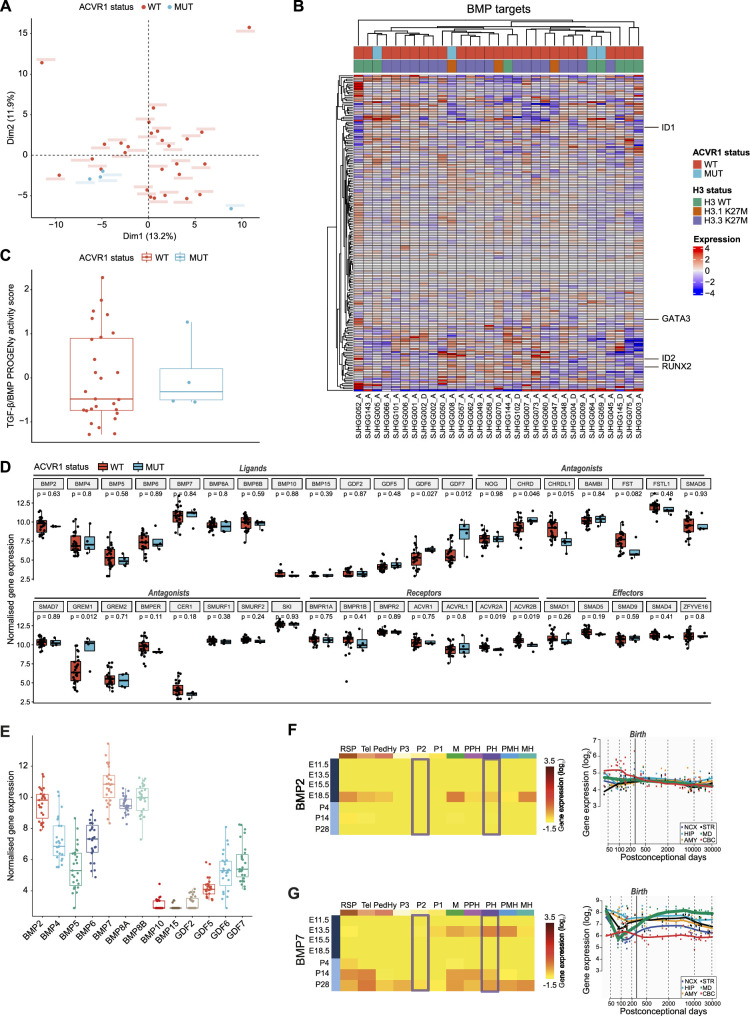
Bone morphogenetic protein (BMP) pathway activation coincides with high BMP2/7 levels in *ACVR1* WT-H3.3K27M pDMG. (**A**) Principal component analysis (PCA) of *ACVR1* WT (red) and mutant pediatric diffuse midline gliomas (pDMG) (blue) samples based on transcriptomic data from cohort 1. (**B**) Heatmap representing the transcriptomic expression levels of BMP target genes between *ACVR1* WT (red) and mutant pDMG (blue) in cohort 1. H3 mutational status is specified as wild-type (WT) (green), mutated on variant H3.1 (brown) or H3.3 (purple). Normalized and centered gene expression levels are color-coded with a blue (low expression) to red (high expression) gradient. Samples in columns are clustered using Euclidean distance. The BMP targets gene list is presented in Supplementary file 1a. (**C**) TGF-β/BMP pathway activity inferred from a specific genes-response signature using PROGENy algorithm in *ACVR1* WT (red) versus mutant pDMG (blue) (cohort 1). No significant difference was observed between both groups. (**D**) Comparison of the expression level of BMP ligands, antagonists, receptors, and effectors between *ACVR1* WT (red) versus mutant pDMG (blue) in cohort 1. p-values are indicated for each gene. (**E**) Boxplot of BMP ligands expression (vst-normalized) in cohort 1. (**F–G**) Pattern of expression of BMP2 and 7 in developing brain. **Left panel:** heatmaps showing relative BMP2 (**F**) and BMP7 (**G**) expression by in situ hybridization (ISH) in murine brain across development obtained from the ALLEN Developing Mouse Brain Atlas. Normalized and scaled gene expression levels are color-coded with a yellow (low expression) to red (high expression) gradient. Developmental stage is mentioned in rows with pre- and post-natal stages color-coded in dark and light blue, respectively. Different brain regions are indicated in columns as follows: RSP: rostral secondary prosencephalon; Tel: telencephalic vesicle; PedHy: peduncular caudal hypothalamus; P3: prosomere 3; P2: prosomere 2; P1: prosomere 1; M: midbrain; PPH: prepontine hindbrain; PH: pontine hindbrain; PMH: pontomedullary hindbrain; MH: medullary hindbrain (medulla). **Right panel:** spatiotemporal gene expression data of BMP2 (**F**) and BMP7 (**G**) expression from human developing and adult brain samples obtained from the Human Brain Transcriptome (Hino et al., 2015). The vertical line indicates birth at 266 days. Each curve represents a part of the brain as following: NCX: neocortex (dark blue); HIP: hippocampus (light blue); AMY: amygdala (orange); STR: striatum (black); MD: mediodorsal nucleus of the thalamus (green, in bold); CBC: cerebellar cortex (red).

Oncogenic (Carvalho et al., 2019; Fortin et al., 2020; Hoeman et al., 2019; Jessa et al., 2022) and tumor-suppressive (Sun et al., 2022) functions of the BMP pathway have both been reported in pDMG. To clarify this point, we first compared tumor clustering according to their *ACVR1* status, based on the transcriptional profiling of 193 BMP pathway target genes as a reliable readout of the pathway activation state, including notably the downstream transducers *ID1*, *ID2*, *RUNX2,* and *GATA3* (see list in Supplementary file 1a). Principal component analysis (PCA) performed on this BMP target genes subset demonstrates no segregation of *ACVR1* mutant (in blue) from *ACVR1* WT samples (in red) in two independent publicly available transcriptomic cohorts of 31 (McLeod et al., 2021) and 40 (Mackay et al., 2017) patients (Figure 1A and Figure 1—figure supplement 1A, respectively). Consistently, the expression pattern of these genes does not segregate pDMG according to their *ACVR1* status in an unsupervised hierarchical clustering analysis in both cohorts (Figure 1B and Figure 1—figure supplement 1B). To have a more comprehensive overview of BMP pathway activation level, we used the PROGENy method to infer downstream TGF-β/BMP response footprint from perturbation-response genes indicative of the pathway activity (Schubert et al., 2018). Interestingly, we observed no significant difference in the TGF-β/BMP activation PROGENy score between *ACVR1* mutant and WT tumors (Figure 1C and Figure 1—figure supplement 1C). Of note, although variable, this score was even higher than the maximum observed in *ACVR1* mutant tumors in 12.5 (cohort 2)–18.5% (cohort 1) of *ACVR1* WT pDMG.

**Figure 1—figure supplement 1.**
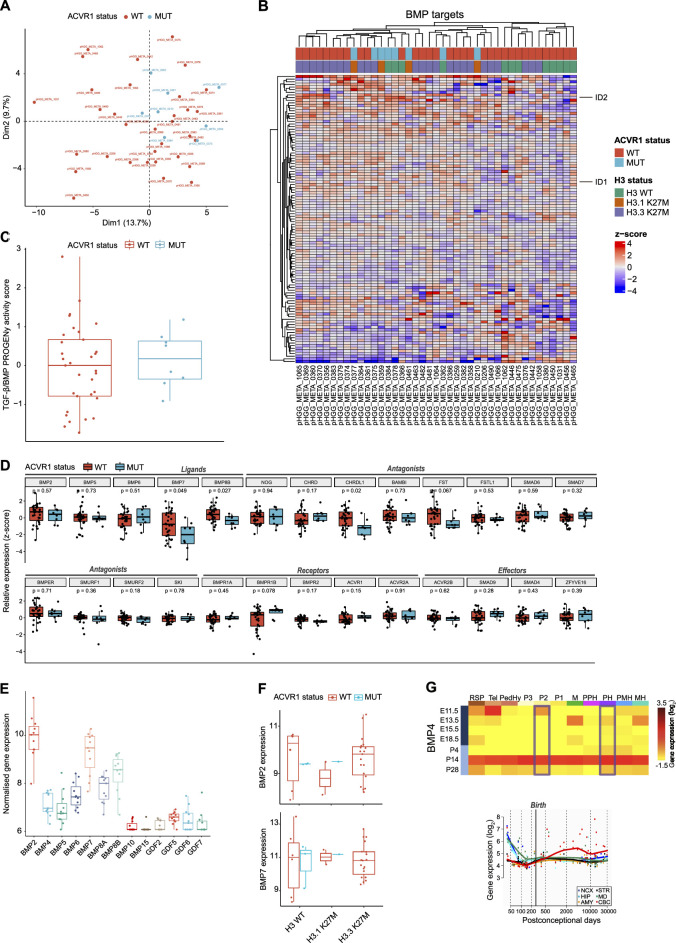
Bone morphogenetic protein (BMP) pathway activation coincides with high BMP2/7 levels in *ACVR1* WT-H3.3K27M pDMG. (**A**) Principal component analysis (PCA) of *ACVR1* WT (red) and mutant pediatric diffuse midline gliomas (pDMG) (blue) samples based on transcriptomic data from cohort 2. (**B**) Heatmap representing the transcriptomic expression levels of BMP target genes between *ACVR1* WT (red) and mutant pDMG (blue) in cohort 2. H3 mutational status is specified as wild-type (WT) (green), mutated on variant H3.1 (brown) or H3.3 (purple). Normalized and centered gene expression levels are color-coded with a blue (low expression) to red (high expression) gradient. Samples in columns are clustered using Euclidean distance. The BMP targets gene list is presented in the Supplementary file 1a. (**C**) TGF-β/BMP pathway activity inferred from a specific genes-response signature using PROGENy algorithm in *ACVR1* WT (red) versus mutant pDMG (blue) (cohort 2). No significant difference was observed between both groups. (**D**) Comparison of the expression levels of BMP ligands, antagonists, receptors, and effectors between *ACVR1* WT (red) versus mutant pDMG (blue) in cohort 2. p-values are indicated for each gene. (**E**) Boxplot of BMP ligands expression (vst-normalized) in cohort 3. (**F**) Boxplot of BMP2 and 7 expressions (vst-normalized) in cohort 1, according to *ACVR1* and *H3* status. (**G**) Pattern of expression of BMP4 in developing brain. **Left panel:** heatmaps showing relative BMP4 expression by in situ hybridization (ISH) in murine brain across development obtained from the ALLEN Developing Mouse Brain Atlas. Normalized and scaled gene expression levels are color-coded with a yellow (low expression) to red (high expression) gradient. Developmental stage is mentioned in rows with pre- and post-natal stages color-coded in dark and light blue, respectively. Different brain regions are indicated in columns as follows: RSP: rostral secondary prosencephalon; Tel: telencephalic vesicle; PedHy: peduncular caudal hypothalamus; P3: prosomere 3; P2: prosomere 2; P1: prosomere 1; M: midbrain; PPH: prepontine hindbrain; PH: pontine hindbrain; PMH: pontomedullary hindbrain; MH: medullary hindbrain (medulla). **Right panel:** spatiotemporal gene expression data of BMP4 expression from human developing and adult brain samples obtained from the Human Brain Transcriptome (Hino et al., 2015). The vertical line indicates birth at 266 days. Each curve represents a part of the brain as following: NCX: neocortex (dark blue); HIP: hippocampus (light blue); AMY: amygdala (orange); STR: striatum (black); MD: mediodorsal nucleus of the thalamus (green, in bold); CBC: cerebellar cortex (red).

To define what alternative mechanism could lead to the activation of the BMP pathway in *ACVR1* WT tumors, we profiled the expression of all major BMP pathway components. Compatible with BMP signaling being active, several ligands, receptors, antagonists and intracellular transducers are expressed in *ACVR1* WT pDMG, albeit at expression levels very similar to those observed in *ACVR1* mutant tumors (Figure 1B and D and Figure 1—figure supplement 1B, D). By differential analysis, we found that only *CHRDL1* is significantly overexpressed in *ACVR1* WT tumors compared to mutant ones in two independent transcriptomic cohorts, as previously reported in pDMG cell lines (Sun et al., 2022; Figure 1D and Figure 1—figure supplement 1D). Although its role in regulating the BMP pathway remains to be fully elucidated (Nakayama et al., 2001; Sakuta et al., 2001; Larman et al., 2009; Blanco-Suarez et al., 2018; Lin et al., 2005), the gain of *CHRDL1* expression is unlikely promoting the activation of the BMP pathway in *ACVR1* WT tumors. Since no difference is observed in receptor expression consistently between the two cohorts (Figure 1D and Figure 1—figure supplement 1D), we hypothesized that activation of this pathway likely results from tumor-autonomous and/or microenvironment-driven production of ligands. Out of all BMP ligands present in pDMG tumors, BMP2 and BMP7 are the two most highly expressed ligands in H3.3K27M pDMG in both cohorts with no significant difference according to H3 and ACVR1 mutational status (Figure 1E and Figure 1—figure supplement 1E, F). Once induced, BMP ligand production can be maintained by a positive transcriptional regulatory loop (Kozmikova et al., 2013; Christiaen et al., 2010). We then reasoned that in *ACVR1* WT pDMG, the priming signal may come from the microenvironment. Accordingly, it has been recently suggested that H3.3K27M DIPG likely occur in cells derived from dorsal PAX3+ BMP-reliant progenitors, and that the oncogenic transformation may result from a crosstalk with BMP ligands present in the microenvironment at that time (Jessa et al., 2022). Regulation of dorsal glial cell fate during development has been shown to rely mostly on BMP4 and BMP7 (Liem et al., 1995; Wilson et al., 2000; Hawley et al., 1995; Wilson and Hemmati-Brivanlou, 1995). By analyzing data from the ALLEN Developing Mouse Brain Atlas (Lein et al., 2007), we observed that BMP7 is only expressed at E13.5 during embryogenesis. It is then progressively re-expressed post-natally from P14 notably in the pontine hindbrain, to reach a maximum in most territories including prosomere 2 at P28, which is compatible with the spatio-temporal window of occurrence of H3.3K27M pDMG tumors (Jessa et al., 2022; Figure 1G). BMP2 is only expressed at the E18.5 embryonic stage and BMP4 expression peaks at P14 corresponding to infancy (Burford, 2021), before decreasing during the post-natal period in these territories (Figure 1F; Figure 1—figure supplement 1G). Similarly, by integrating transcriptomic data from the HBT program (Kang et al., 2011), we observed that BMP7 expression but not BMP2 and BMP4 gradually increases to reach its maximum in the midline structure in the mid-childhood period (green curve, Figure 1F, G and Figure 1—figure supplement 1G), then coinciding with the peak incidence of pDMG tumors (Mackay et al., 2017).

Overall, the integration of transcriptomic data reveals that the induction of BMP signaling in *ACVR1* WT pDMG, at a level equivalent to that observed in mutant tumors, could notably result from the production of BMP2 and/or 7, initiated by the expression of BMP7 by the microenvironment.

### BMP7 synergizes with K27M to induce a transcriptomic program leading to quiescence and invasiveness in a low-grade glioma model

**Figure 2.**
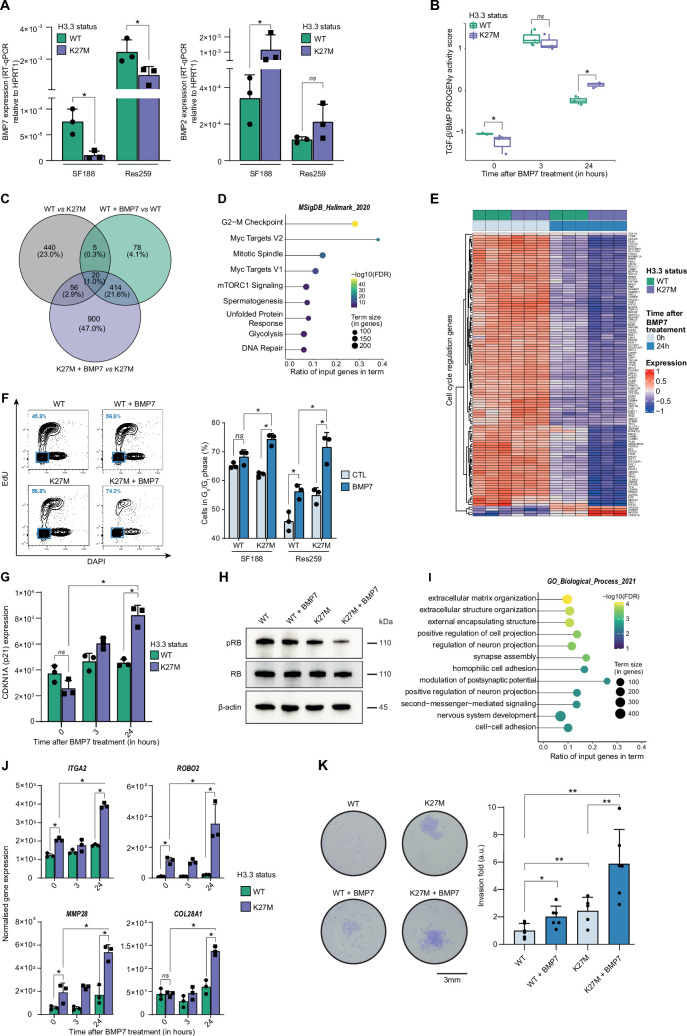
BMP7 induces a specific transcriptomic and phenotypic switch in a H3.3K27M mutant glioma context. (**A**) *BMP7* (left) and *BMP2* (right) expressions in H3.3WT (green) versus H3.3K27M (purple) SF188 and Res259 cells. Gene expressions were analyzed by qRT-PCR relative to *HPRT1* expression. Means ± std are represented (n=3). * p<0.05, ns: non-significant. (**B**) TGF-β/BMP pathway activity inferred from a specific genes-response signature using the PROGENy algorithm in Res259-H3.3WT (green) and H3.3K27M (purple) after 0, 3 and 24 hr of BMP7 treatment (n=3). *p<0.05, ns: non-significant. (**C**) Venn diagram showing the number of differentially expressed genes (DEG) and the corresponding percentages compared to all DEG in each condition: Res259-H3.3WT versus Res259-H3.3K27M without BMP7 treatment (grey), Res259-H3.3WT versus Res259-H3.3WT treated with BMP7 for 24 hr (green), Res259-H3.3K27M versus Res259-H3.3K27M treated with BMP7 for 24 hr (purple). (**D**) Functional enrichment of DEG specifically between Res259-H3.3K27M versus Res259-H3.3K27M treated with BMP7 for 24 hr. Dots are colored according to their false discovery rate with a blue (lower significance) to yellow (higher significance) gradient and sized by the count number of genes matching the biological process. (**E**) Heatmap representing the transcriptomic expression levels of genes associated with cell cycle regulation between Res259-H3.3WT (green) and Res259-H3.3K27M (purple) cells, with (dark blue) or without (light blue) BMP7 treatment. Normalized and centered gene expression levels are color-coded with a blue (low expression) to red (high expression) gradient. (**F**) Flow cytometry analyses of cell cycle in Res259/SF188-H3.3WT and H3.3K27M upon BMP7 treatment. **Left panel:** representative density plots with outliers (dots) with 5-ethynyl-2′-deoxyuridine (EdU) staining on the y-axis and with DAPI staining on the x-axis for the indicated conditions on Res259 cell lines. Quantification of cells in G0/G1 phase (blue square, low EdU, and low DAPI stainings) appear in the lower left corner for the presented graph. **Right panel:** quantification of cells in G0/G1 phase for SF188- and Res259-H3.3WT or H3.3K27M without BMP7 treatment (light blue) or after 24 hr treatment (dark blue). Means ± std are represented (n=3). *p<0.05, ns: non-significant. (**G**) *CDKN1A* (encoding p21) normalized expression from transcriptomic data of Res259-H3.3WT (green) and Res259-H3.3K27M (purple) after 0, 3, or 24 hr of BMP7 treatment. Means ± std are represented (n=3). *p<0.05, ns: non-significant. (**H**) Western-blot analysis of RB phosphorylation on S780 (pRB) in Res259-H3.3WT or H3.3K27M upon BMP7 treatment. Total RB and β-actin are used as controls. One representative experiment out of 3 is shown. (**I**) Functional enrichment of DEG specific for the K27M/BMP7 condition, according to the decision tree algorithm presented in Figure 2—figure supplement 1J. Dots are colored according to their false discovery rate with a blue (lower significance) to yellow (higher significance) gradient and sized by the count number of genes matching the biological process. (**J**) *ITGA2*, *ROBO2*, *MMP28,* and *COL28A1* normalized expression from transcriptomic data of Res259-H3.3WT (green) and Res259-H3.3K27M (purple) after 0, 3 or 24 hr of BMP7 treatment. Means ± std are represented (n=3). *p<0.05, ns: non-significant. (**K**) Impact of BMP7 treatment on invasion in Res259-H3.3WT versus H3.3K27M. **Left panel:** representative pictures of a transwell invasion assay of Res259-H3.3WT or H3.3K27M, with and without BMP7 treatment. Scale bar = 3 mm. **Right panel:** invasion was quantified as the mean value of five independent experiments and represented as a graph. Means ± std are represented. *p<0.05, **p<0.01. Figure 2—source data 1.Uncropped and labeled gels for Figure 2. Figure 2—source data 2.Raw unedited gels for Figure 2.

To functionally dissect the impact of H3.3K27M/BMP crosstalk and define whether it may have a rather oncogenic or tumor suppressive value, we first used the two previously described pediatric glioma Res259 and SF188 cell lines, which have been genetically modified to stably express and reproduce the epigenetic context either of WT or mutated forms of the variant H3.3 (Rakotomalala et al., 2021). Interestingly, BMP7 expression significantly decreases after H3.3K27M induction in both SF188 and Res259 cells (Figure 2A), whereas BMP2 and BMP4 expressions are respectively increased or unmodified (Figure 2A and Figure 2—figure supplement 1A, B). To compensate for that decrease and mimic the high level of BMP7 expression observed in pDMG tumors, we then assessed the impact of recombinant BMP7 depending on the H3.3 context.

**Figure 2—figure supplement 1.**
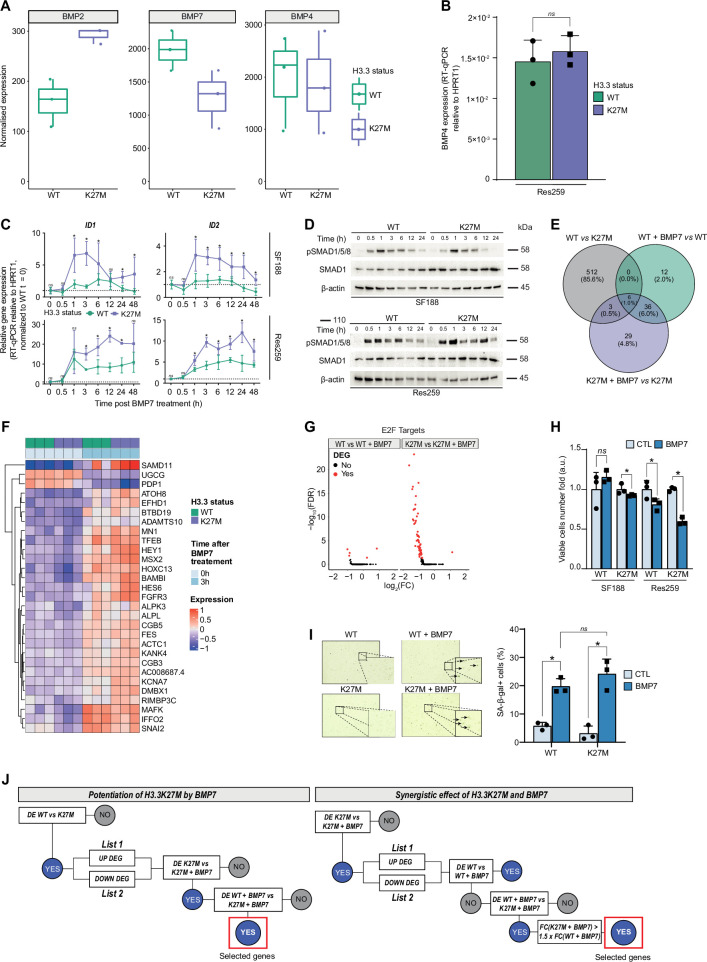
H3.3K27M mutant context potentiates BMP7-induced transcriptomic and phenotypic switch in a glioma model. (**A**) *BMP2*, *BMP7,* and *BMP4* normalized expression from transcriptomic data of Res259-H3.3WT (green) *versus* Res259-H3.3K27M (purple). Means ± std are represented (n=3). (**B**) *BMP4* expression between Res259-H3.3WT (green) and H3.3K27M (purple). Gene expression was analyzed by qRT-PCR relative to *HPRT1* expression. Means ± std are represented (n=3). ns: non-significant p-value. (**C**) *ID1* and *ID2* expressions between SF188- (upper panel) and Res259- (bottom panel) H3.3WT (green) and H3.3K27M (purple) following BMP7 treatment at indicated time points from 0 to 48 hr (x-axis). Means ± std are represented (n=3). *p< 0.05, ns: non-significant. (**D**) Western-blot analysis of SMAD1/5/8 phosphorylation on Ser463/465 (SMAD1/5) and Ser465/467 (SMAD8) (pSMAD1/5/8) levels in SF188- and Res259-WT and H3.3K27M upon BMP7 treatment. Total SMAD1 and β-actin are used as loading control for the indicated conditions (n=3). A representative experiment is shown. (**E**) Venn diagram showing the number of differentially expressed genes (DEG) and the corresponding percentages compared to all DEG in each condition: Res259-H3.3WT versus Res259-H3.3K27M without BMP7 treatment (grey), Res259-H3.3WT versus Res259-H3.3WT treated with BMP7 for 3 hr (green), Res259-H3.3K27M versus Res259-H3.3K27M treated with BMP7 for 3 hr (purple). (**F**) Heatmap representing the transcriptomic expression levels of the 29 DEG specifically in Res259-H3.3K27M (n=3) versus Res259-H3.3K27M treated with BMP7 for 3 hr (n=3). Samples are placed in columns such as Res259-H3.3WT (green) and -H3.3K27M (purple), control (light blue), or 3 hr BMP7 treatment (blue). Normalized and centered gene expression levels are color-coded with a blue (low expression) to red (high expression) gradient. (**G**) Volcano plots of DEG in the E2F target signature between Res259-H3.3WT control *versus* BMP7-treated (left panel) and Res259-H3.3K27M control *versus* BMP7-treated (right panel). Fold change is indicated on the x-axis and statistical significance on the y-axis. Black dots: non-differentially expressed. Red dots: differentially expressed. (**H**) Viable cell count of SF188- and Res259-H3.3WT and H3.3K27M after 72 hr of BMP7 treatment (dark blue), normalized to non-treated condition (light blue). Means ± std are represented (n=3). *p< 0.05, ns: non-significant. (**I**) Senescence-associated beta-galactosidase activity (SA-β-gal) assessment experiment on Res259-H3.3WT and Res259-H3.3K27M control or treated with BMP7 for 72 hr with SA-β-gal staining. Right panel: representative microscopic photos. Arrows indicate SA-β-gal+ cells. Left panel: quantification of cells positive for SA-β-gal activity. Means ± std are represented (n=3). *r< 0.05, ns: non-significant. (**J**) Decisional tree algorithm to study the specific impact of potentiation of H3.3K27M by BMP7 (left) and the synergy of both (right). DE: differentially expressed. DEG: differentially expressed genes. FC: fold-change. K27M: H3.3K27M mutation. WT: H3.3wt. For the last step of decisional tree 2 (right panel), genes are selected such as log2(FC(K27M+BMP7)/(K27M))>1.5 x log2(FC(WT + BMP7)/(WT)). Figure 2—figure supplement 1—source data 1.Uncropped and labeled gels for Figure 2—figure supplement 1. Figure 2—figure supplement 1—source data 2.Raw unedited gels for Figure 2—figure supplement 1.

Using qRT-PCR as a first hint, we observed a significant increase in BMP targets expression following BMP7 treatment in both H3.3K27M-Res259 and -SF188 cells compared to H3.3 WT cell lines, with at least a twofold increase at 3 and 24 hr for both *ID1* and *ID2* (Figure 2—figure supplement 1C). Interestingly, both levels and kinetics of SMAD1/5/8 phosphorylation in H3.3K27M versus WT overexpressing cells remain unchanged, indicating that BMP canonical pathway activation is not modified by histone mutational status (Figure 2—figure supplement 1D). To further investigate and characterize the specificity of the response of H3.3 WT and mutant cells to BMP7, we performed an RNA-sequencing (RNA-seq) analysis after 3 hr and 24 hr of treatment with recombinant BMP7. Using PROGENy analysis, we observed that the increase in TGF-β/BMP activation score induced by BMP7 is potentiated and remains significantly higher at 24 hr in H3.3K27M-Res259 compared to their wild-type counterparts (Figure 2B). While differentially expressed genes at 3 hr mostly correspond to the expression of the K27M mutation (Figure 2—figure supplement 1E, F), a subset of 900 genes appears differentially expressed (DE) specifically between treated and untreated H3.3K27M-Res259 cells, but not in H3.3 WT ones (Figure 2C). Enrichment analyses revealed that DE genes are notably associated with alteration in cell cycle regulation (Figure 2D–E), and that the downregulated ones in H3.3K27M BMP7-treated cells correspond to E2F targets (Figure 2—figure supplement 1G). Consistently, BMP7 treatment leads to a significant 12.2% and 16.7% increase of cells in the G0/G1 phase, respectively in H3.3K27M-SF188 and -Res259 cells, while its effect is limited to a 3% and 10.3% increase in their WT counterparts (Figure 2F). Similarly, the number of H3.3K27M-Res259 cells is significantly reduced by 1.7-fold compared to non-treated cells upon BMP7 treatment, while the decrease is limited to 1.2-fold in WT ones (Figure 2—figure supplement 1H). Of note, this K27M-dependent BMP7 effect is associated with a significant 1.8-fold increase in cyclin dependent kinase inhibitor 1 A (*CDKN1A*, encoding P21; Figure 2G) expression and a reciprocal decrease in RB1 phosphorylation (Figure 2H). This cell cycle blockade is unlikely to result from the entry of cells in senescence since there is no difference in beta-galactosidase activity (SA-β-gal) between wild-type and mutant cells upon BMP7 treatment (Figure 2—figure supplement 1I).

To further dissect the crosstalk between BMP7 and the H3.3K27M mutation, we established a decision tree algorithm to specifically isolate which of the 900 DE genes post-treatment BMP7 in H3.3K27M cells correspond to a potentiation of the effect of the K27M mutation by BMP7 (Figure 2—figure supplement 1J, left panel), or to a specific effect of BMP7 in the K27M context (Figure 2—figure supplement 1J, right panel). We then pinpointed DE genes that specifically correspond to cooperative effects of K27M epigenetic alterations and BMP7-mediated transcriptional regulation. Interestingly, enrichment analyses revealed that these genes are involved in processes related to invasion/migration, including extracellular matrix organization, regulation of cell/neuron projection, and adhesion (Figure 2I). Some of these genes such as *ITGA2*, *ROBO2*, and *MMP28* are already induced following H3.3K27M expression, and their expression is further amplified by the BMP7 treatment (Figure 2J). Conversely, others, such as *COL28A1,* are specifically induced or repressed by the K27M+BMP7 context (Figure 2J), consistently with our decision tree algorithm. We then assessed the combined impact of H3.3K27M expression and exposure to BMP7 on the invasive properties of glioma cells using a Matrigel-coated transwell assay (Figure 2K). Expression of the H3.3K27M mutation is sufficient to drive a moderate increase in invasion compared to the WT context. However, this phenomenon is largely amplified by BMP7, with a global 5.6-fold in the H3.3 mutant context, compared to twofold in the H3.3 WT one.

Altogether, these data support the fact that BMP7 is sufficient to induce a transcriptomic reprogramming specific to the H3.3K27M epigenetic context, which leads to the emergence of a quiescent but invasive cell state.

### Combined BMP2/BMP7 expression drives a quiescent-invasive tumor cell state in pDMG

**Figure 3.**
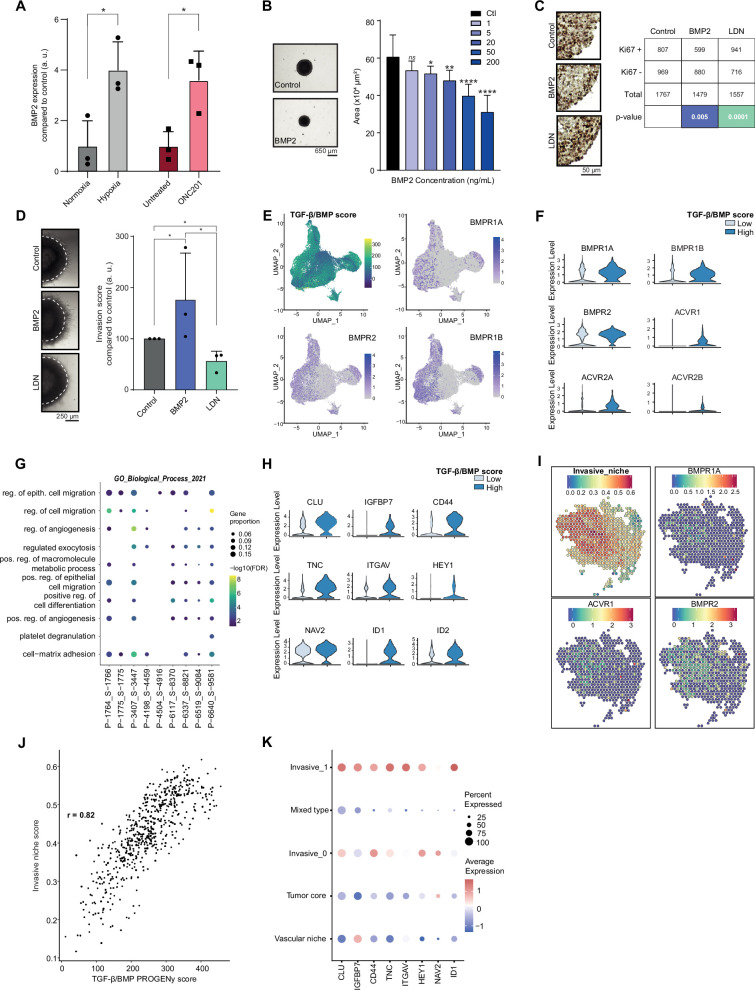
Combined tumor-autonomous BMP2/BMP7 expression drives a quiescent-invasive tumor cell state in pediatric diffuse midline gliomas (pDMG). (**A**) *BMP2* expression after hypoxia or ONC201 treatment. Gene expression was analyzed by QRT-PCR relative to *HPRT1* expression. Means ± std are represented (n=3). *p<0.05. (**B**) Growth monitoring of HSJD-DIPG-012 following recombinant BMP2 treatment. Means± std are represented (n=3). *p<0.05, **p<0.01, ****p<0.0001, ns: non-significant. Scale bar = 650µm. (**C**) Impact of BMP2 or LDN treatment on KI67-positive staining in HSJD-DIPG-012. **Left panel:** representative images of Ki67 immunohistochemistry on HSJD-DIPG-012 spheroids treated or not with either BMP2 or LDN-193189. Scale bar = 50 µm. **Right panel:** quantification of Ki67-positive and negative cells. p-values were computed using Fisher’s exact test. BMP2: 200 ng/mL, LDN-193189: 1 µM. (**D**) Impact of BMP2 or LDN treatment on tumor cells invasion. **Left panel:** representative images of HSJD-DIPG-012 spheroids embedded in Matrigel, after 48 hr of BMP2 or LDN-193189 treatment. Scale bar = 250 µm. **Right panel:** Invasion was quantified as the mean value of four independent experiments and represented as a graph. *p<0.05. BMP2: 10 ng/mL, LDN-193189: 1 µM. (**E**) UMAP (uniform manifold approximation and projection) computed on harmony embeddings of the tumor cells of 10 *ACVR1* WT-H3.3K27M pDMGs from the scRNA-seq data published by Jessa et al., 2022. PROGENy TGF-β/BMP score is colored from blue (low activity score) to yellow (high activity score). Expression of the bone morphogenetic protein (BMP) receptors *BMPR1A*, *BMPR2* and *BMPR1B* is colored from grey (low expression values) to purple (high expression values). (**F**) Violin plots of BMP receptors in one (P-1775_S-1775) of the 10 samples based on the TGF-β/BMP PROGENy score. The TGF-β/BMP-low and high groups are colored respectively in light and dark blue. All p-value are lower than 7.357206e-13 (*ACVR2A*). (**G**) Dotplot of the first 10 significantly enriched pathways (FDR ≤ 0.05) in TGF-β/BMP-high cells for each of the 10 *ACVR1* WT-H3.3K27M pDMGs, ranked by number of samples with a significant enrichment, using the GO Biological Process database. Only dots of significant enrichments are shown for each sample. Dot color represents the -log10 (p-value) and ranges from blue (high p-value) to yellow (low p-values). Dot size is proportional to the overlap of differentially expressed (DE) genes and the genes of a geneset. (**H**) Violin plots of invasion-related genes in one (P-3407_S-3447) of the 10 samples based on the TGF-β/BMP-high/low score. The TGF-β/BMP-low group and high group are respectively colored in light and dark blue. All p-value are lower than 1.34e-8 (*HEY1*). (**I**) ‘Invasive niche’ score from Ren et al., 2023, and associated expression of the BMP receptors *BMPR1A*, *ACVR1,* and *BMPR2*. Color ranges from blue (low score/expression value) to red (high score/expression value). (**J**) Scatter plot correlating the PROGENy TGF-β/BMP pathway activity score with the ‘Invasive niche’ score for each Visium spot of pDMG Sample-1. The correlation coefficient was computed using Pearson’s method. ****p-value < 2.e-16. (**K**) Scaled expression of invasion-related genes in pDMG Sample-1 for each identified area. Dot size represents the proportion of cells expressing the gene. Color ranges from blue (low expression) to red (high expression).

Considering the data obtained in the Res259/SF188 mechanistic models, we then sought to define whether this crosstalk between BMP and H3.3K27M was preserved with similar effects in H3.3K27M pDMG models. First, we observed that BMP7 is the most expressed ligand in two *ACVR1* WT/H3.3K27M DIPG cell lines, but that few if any other BMP ligands, including BMP4, are (Figure 3—figure supplement 1A). Unlike in tumors, BMP2 expression is notably low in these cell lines (Figure 3—figure supplement 1A). BMP2 has been shown to be induced by hypoxia (Tseng et al., 2010) or reactive oxygen species (ROS) (Sánchez-de-Diego et al., 2019). Accordingly, exposure of *ACVR1* WT H3.3K27M DIPG cells to hypoxia or ONC201, which is known to significantly increase ROS production (Przystal et al., 2022; Wu et al., 2023), are both sufficient to induce a significant increase in BMP2 expression (Figure 3A). Thus, BMP2 can be produced autonomously by tumor cells in response to specific stresses. To model the concomitant impact of BMP7 and stress-induced BMP2 in the H3.3K27M mutant background, we analyzed the impact of BMP2 addition on DIPG 3D spheroids. As shown in Figure 3B and Figure 3—figure supplement 1B, C, treatment of DIPG spheroids with increasing doses of recombinant human BMP2 triggers SMAD1/5/8 phosphorylation and leads to a strong dose-dependent decrease in growth rate. Consistently, Ki67 staining, a marker of proliferation, is significantly decreased upon BMP2 treatment (Figure 3C). Reciprocally, treatment of DIPG-spheroids with the BMP inhibitor LDN-193189 (LDN) leads to a slight increase in KI67-positive cells (Figure 3C). This effect was largely mitigated in BT245 and DIPGXIII cell lines in which the K27M mutant allele was removed (Harutyunyan et al., 2019), indicating that it may depend on the K27M-specific epigenetic context (Figure 3—figure supplement 1D). Of particular interest, the combined knock-out of K27M and BMP inhibition with LDN treatment resulted in the failure of pDMG cells to proliferate and form gliospheres (Figure 3—figure supplement 1D). In parallel, we explored the impact of BMP activation/inhibition on DIPG cells migration/invasion propensity. BMP2 significantly increases the migration of tumor cells from Matrigel-embedded 3D-DIPG spheroids, while an antagonistic effect was observed upon LDN treatment (Figure 3D and Figure 3—figure supplement 1E).

**Figure 3—figure supplement 1.**
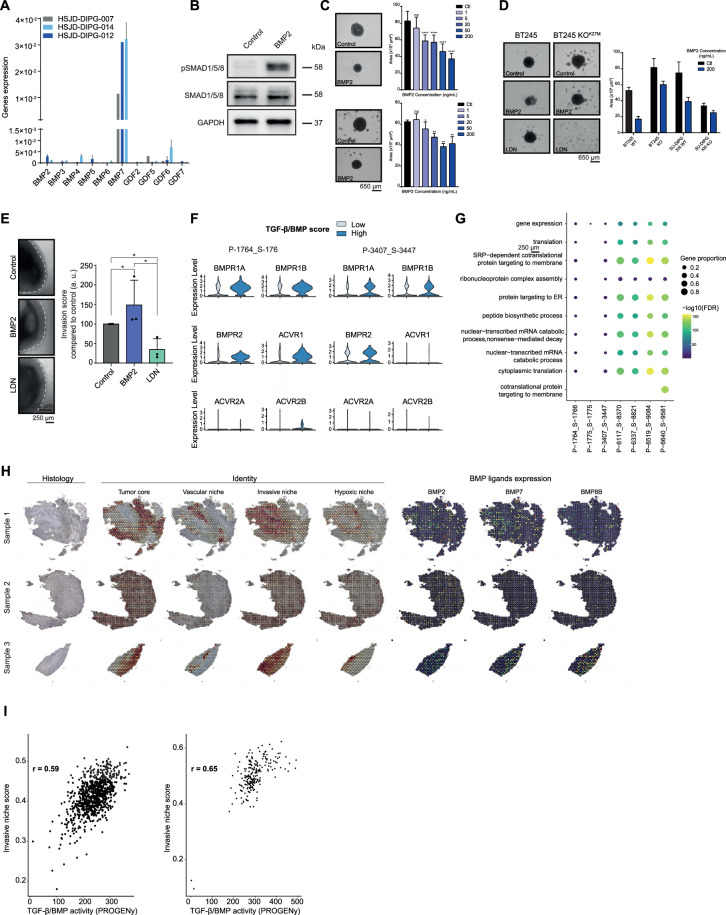
Combined tumor-autonomous BMP2/BMP7 expression drives a quiescent-invasive tumor cell state in pediatric diffuse midline gliomas (pDMG). (**A**) Bone morphogenetic protein (BMP) ligands expression analyzed by qRT-PCR relative to five housekeeping genes levels in three different Diffuse Intrinsic Pontine Gliomas (DIPG) cell lines. HSJD-DIPG-007: *ACVR1* mutant (grey). HSJD-DIPG-012: *ACVR1* WT (dark blue). HSJD-DIPG-014: *ACVR1* WT (light blue). Means ± std are represented (n=2). (**B**) Western-blot analysis of pSMAD1/5/8, total SMAD1/5/8 on HSJD-DIPG-012 spheroids, treated or not with BMP2. GAPDH was used as a loading control. A representative experiment out of 3 is shown. (**C**) Growth monitoring of HSJD-DIPG-014 (top panel) and HSJD-DIPG-013 (bottom panel) following recombinant BMP2 treatment. Means ± std are represented. *p<0.05, **p<0.01, ***p<0.001. Scale bar = 650µm. (**D**) Growth monitoring of BT245 and DIPGXIII, before and after (KO^K27M^) edition of the H3.3K27M mutation. Representative pictures of BT245 are shown on the left. Means ± std are represented on the right. Scale bar = 650µm. (**E**) Impact of BMP2 or LDN treatment on tumor cells invasion. **Left panel:** representative images of HSJD-DIPG-013 spheroids embedded in Matrigel, after 48 hr of BMP2 or LDN-193189 treatment. Scale bar = 250 µm. **Right panel:** Invasion was quantified as the mean value of four independent experiments and represented as a graph. *p<0.05. BMP2: 10 ng/mL. LDN:1 µM. (**F**) Violin plots of BMP receptors in two (P-1764_S-1766 and P-3407_S-3447) of the 10 samples based on the TGF-β/BMP score. The TGF-β/BMP-low group is colored in light blue and the TGF-β/BMP-high group is colored in blue. (**G**) Dotplot of the first 10 significantly enriched pathways (FDR ≤ 0.05) in TGF-β/BMP-low cells for each of the 10 *ACVR1* WT-H3.3K27M pDMG, ranked by number of samples with a significant enrichment, using the GO Biological Process database. Only dots of significant enrichments are shown for each sample. Dot color represents the -log10(p-value) and ranges from blue (high p-value) to yellow (low p-values). Dot size is proportional to the overlap of differentially expressed (DE) genes and the genes of a geneset. (**H**) Score of the pDMG niche signatures identified by Ren et al., 2023, namely: ‘Tumor core,’ ‘Vascular niche,’ ‘Invasive niche’ and ‘Hypoxic niche’ in each of the three Visium samples. Color ranges from blue (low score value) to red (high score value). (**I**) Scatter plots correlating PROGENy TGF-β/BMP pathway activity with the ‘Invasive niche’ score for each Visium spot of Sample-2 and Sample-3. The correlation coefficient was computed using Pearson’s method. ****p-value < 2.e-16. Figure 3—figure supplement 1—source data 1.Uncropped and labeled gels for Figure 3—figure supplement 1. Figure 3—figure supplement 1—source data 2.Raw unedited gels for Figure 3—figure supplement 1.

To define whether such a BMP-induced quiescent-invasive cell state exists in *ACVR1* WT H3.3K27M pDMG tumors, we first used publicly available scRNA-seq data from 10 patients’ biopsies from Jessa et al., 2022. Integration of all these data unveiled that the BMP-responsive (i.e. High PROGENy TGF-β/BMP score) pool of tumor cells correlates significantly with BMP receptors expression, in particular to *BMPR2*, *BMPR1B*, *BMPR1A*, and *ACVR1* levels, respectively in 8, 5, 5, and 4 out of 10 samples (Figure 3E–F and Figure 3—figure supplement 1F). Interestingly, this BMP-responsive pool of cells is significantly enriched in genes involved in positive regulation of cell migration and in cell-matrix adhesion (Figure 3G), while showing a specific quiescent-compatible decrease in genes involved in transcription/translation processes (Figure 3—figure supplement 1G). To further define the extent and organization of this pool of cells, we performed spatial transcriptomic analysis of 3 H3.3K27M pDMG tumors. Data integration was performed using pDMG-derived Visium and scRNA-seq signatures recently published by Ren et al., 2023 and Filbin et al., 2018 (Figure 3—figure supplement 1H). Consistently with observations in bulk and single-cell analyses of patients' tumors/models, the most highly expressed BMP ligands are BMP2 and BMP7 along with BMP8B (Supplementary file 1b and Figure 3—figure supplement 1H), whose expression is not spatially delimited or associated with a specific gene signature. In line with single-cell analyses, we observed on the contrary that BMP receptors expression correlate significantly with the invasive niche score defined by Ren et al., 2023 (Figure 3I), which can be spatially restricted to histologically delineated areas (samples 1–3, Figure 3I and Figure 3—figure supplement 1H) or more dispersed within the tumor (sample 2, Figure 3—figure supplement 1H). Moreover, the invasive niche score strongly correlates to the PROGENy TGF-β/BMP score (Figure 3J and Figure 3—figure supplement 1I). Coherently, this BMP-responsive invasive niche is characterized by a high expression of key markers of BMP activity (*ID1*), as well as markers of stemness (*CD44*, *HEY1*) and migration/invasion (*TNC*, *IGFBP7*, *ITGAV*) (Figure 3K).

Altogether, these data indicate that tumor-autonomous production of BMP2 and BMP7 synergize to maintain a quiescent-invasive niche in H3.3K27M DIPG.

## Discussion

Major advances have been made in understanding the molecular bases of pDMG, among which DIPG, with the identification of major epigenetic remodeling processes induced by histone H3 mutations. However, the oncogenic mechanisms cooperating with these mutations to induce transformation and tumor escape remain largely undefined.

Along this line, a key challenge is to establish the precise role of the transcriptomic reprogramming induced by the BMP pathway, which remains controversial in these pediatric brain tumors (Carvalho et al., 2019; Fortin et al., 2020; Hoeman et al., 2019; Jessa et al., 2022; Sun et al., 2022). Herein, we performed a comprehensive integration of transcriptomic data, which first supports the view of BMP signaling being also clearly activated in *ACVR1* WT/H3.3K27M pDMG. Further analyzes will be necessary to define whether the lowest levels of activation correspond to samples with specific locations along the midline. Effect of BMP activation is potentiated by the epigenetic context of these tumors, then leading to a global transcriptional reprogramming. Although it has been previously described that *ACVR1*-mutant tumors exhibit a higher expression of *ID1* and *ID2* (Carvalho et al., 2019), the extrapolation of BMP activity from a larger signature of BMP targets and on a score calculated from the inference of gene expression perturbations in response to the TGF-β/BMP pathway indicates the existence of a compensatory mechanism driving BMP activation in non-*ACVR1* mutant tumors. By analyzing the expression of BMP effectors in pDMG tumors and patient-derived DIPG models, we propose that the activation of this pathway in *ACVR1* WT H3.3K27M tumors could be at least partially mediated by two complementary tumor-autonomous and microenvironment-dependent mechanisms. Upon initiation, the expression of BMP, and notably BMP7, could synergize with or even trigger the autocrine production of BMP ligands by the tumors, among which BMP2 and 7. Indeed, such positive feedback loops maintaining the expression of BMP ligands have already been described notably during development (Kozmikova et al., 2013; Christiaen et al., 2010). The fact that the expression of BMP ligands is similar in *ACVR1* mutant and WT tumors and independent of H3 status (Figure 1—figure supplement 1F) suggests that this regulatory loop is involved in most tumors, but probably by different mechanisms depending on the mutational context. Thus, BMP secretion by the microenvironment may prime the BMP activation in tumor cells and be required for oncogenic transformation. Once established, the dynamic modulation of BMP2 expression in response to stresses, such as hypoxia or treatments, could synergize with constitutive production of BMP7 to drive the emergence of an aggressive cell state. Of note, the expression of BMP-target genes *ID1* to *4* was previously reported to be strongly decreased in pDMG cell lines in which the K27M mutation was removed by CRISPR-Cas9 (Harutyunyan et al., 2019), suggesting that even the maintenance of BMP activation in a pool of tumor cells relies on the specific K27M-mediated epigenetic context. In addition, blocking the BMP pathway with LDN in these K27M-KO cells induces tumor cell death, supporting the view that the K27M/BMP oncogenic synergy plays a major role in maintaining oncogenic potential (Figure 3—figure supplement 1).

Second, our data are in favor of a rather global oncogenic role of the BMP pathway in pDMG gliomagenesis. The tumor suppressor activity of the BMP pathway had been largely extrapolated because of its positive impact on tumor cell quiescence (Sun et al., 2022). Indeed, using a genetically engineered glioma model, we confirmed that BMP7 is sufficient to potentiate the entry of cells in a quiescent state in a H3.3K27M-dependent manner, via a transcriptomic switch largely relying on the downregulation of E2F-targets cell-cycle regulating genes. Nevertheless, this quiescent cellular phenotype could paradoxically constitute an aggressive treatment-resistant state, as previously observed in adult glioblastoma (Atkins et al., 2019; Chen et al., 2012; Xie et al., 2022) and thus explaining its increase in response to treatment (Sun et al., 2022). Along the same line, the impact of *CHRDL1* increase in H3.3K27M tumors on BMP pathway inhibition may probably need to be qualified (Sun et al., 2022). Indeed, if CHRDL1 was first classified as a member of the chordin family of secreted BMP antagonists due to sequence homology, it has been shown that it exerts BMP-independent functions in synapse plasticity and maturation (Blanco-Suarez et al., 2018). Moreover, this protein has also been described as an activator of the BMP pathway (Lin et al., 2005), suggesting that its high level of expression may not be a robust marker of BMP activation state.

However, it cannot be ruled out also that different ligands of the BMP pathway may have different impacts on the cellular phenotype induced by the H3.3K27M mutation. Accordingly, it was shown that BMP4 treatment promotes the differentiation of DIPG tumor cells, in line with its putative tumor suppressor activity (Sun et al., 2022). However, beyond quiescence, we observed that BMP activation by BMP2/7 in a H3.3K27M epigenetic context induces a transcriptomic switch rather than conferring enhanced invasion potential to pDMG tumor cells. Because the level of BMP2/7 is particularly important (i) in bulk, single-cell and spatial transcriptomic tumors, (ii) in patient-derived cell models, and because the dynamics of BMP7 induction in the post-natal period coincide with the spatio-temporal window of pDMG onset, we believe that the role of the BMP2/7 couple is non-negligible in the pathogenesis of pDMG. Interestingly, this pair has already been shown to trigger BMP signaling as a heterodimer notably during embryogenesis, with a higher efficiency than homodimers (Kim et al., 2019; Schmid, 2000; Tajer et al., 2021). Keeping in mind that BMP2 appears to be dynamically regulated by tumor cells upon stress, these data suggest that activation of the BMP pathway may be finely regulated in pDMG to be maintained at optimal pro-oncogenic levels, without triggering the tumor-suppressive effects (Sun et al., 2022) or negative regulatory loops associated with its over-activation (Christiaen et al., 2010; Afrakhte et al., 1998; Akizu et al., 2010; Bénazet et al., 2009).

The next question is whether a therapeutic perspective can be defined by targeting the crosstalk between epigenetic modifications induced by the H3.3K27M mutation and transcriptomic reprogramming induced by BMP2/7. The BMP2/7-driven cell state that we described here fits with previous results obtained in other glioma models, in which a subpopulation of quiescent cells was identified as partially responsible for tumor invasiveness (Atkins et al., 2019; Antonica et al., 2022; Furst et al., 2022), a hallmark of pDMG aggressiveness (Kluiver et al., 2020). Given the complexity of the phenotype induced by H3.3K27M/BMP crosstalk and the pleiotropic role of BMP proteins in the central nervous system, the therapeutic strategy to be developed should be based on the targeting of the downstream effectors, such as ID1 as recently described (Messinger et al., 2023), or TNC, which may be responsible for the invasive phenotype. Upcoming challenges will be to precisely define the identity of these pro-invasive BMP effectors, to set up a combinatorial therapeutic approach, simultaneously targeting the proliferative compartment and the BMP-responsive H3.3K27M invasive cell state.

## Materials and methods

### Gene expression analyzes of publicly available transcriptomic datasets

#### Gene-expression analyzes of H3.3K27M-pDMG transcriptomic cohorts

For cohort 1 (McLeod et al., 2021), HTSeq gene counts and somatic vcf of St Jude’s pDMG samples were downloaded from https://platform.stjude.cloud/. DESeq2 (v 1.36) (Love et al., 2014) was used to normalise the data with the variant stabilization transformation (vst) (Anders and Huber, 2010). *ACVR1* mutation status was assessed using tabix (Li, 2011) (v 1.15.1) to query the region of the *ACVR1* gene chr2:157,736,251–157,876,330 (hg38). Identified variants were manually curated. PROGENy (v 1.18.0) (Schubert et al., 2018) was used to infer TGF-β/BMP pathway activity with the ‘top’ parameter set to 100 (default value).

For cohort 2 (Mackay et al., 2017), gene expression (z-score) and tumor variants were downloaded on https://pedcbioportal.kidsfirstdrc.org/ from the dataset ‘phgg_jones_meta_2017.’ Samples were filtered based on the following location: brainstem or midline, and the following histone mutation: WT, H3.1K27M, and H3.3K27M. Only the four datasets with *ACVR1* mutant samples were kept for the analysis to avoid biases: ‘PMID:21931021|PMID:24705251,’ ‘PMID:21931021|PMID:22286216,’ ‘PMID:22389665|PMID:24705252,’ ‘PMID:24705251.’ TGF-β/BMP pathway activity was also inferred using PROGENy, but the ‘top’ parameter was set to 178 genes: as half of the top 100 genes of the PROGENy model for the TGF-β/BMP pathway are not covered in cohort 2, we identified the top 100 genes with the highest absolute coefficient in PROGENy’s model to compute pathway activity with the same number of genes in both cohorts.

For both cohorts, PCA was performed using FactoMineR (v.2.4) (Lê et al., 2008) and plotted with factoextra (v1.0.7) (Kassambara and Mundt, 2017).

#### Patterning of BMP expression in the developing brain

Spatio-temporal gene expression data of *BMP2*, *BMP4* and *BMP7* from human developing and adult brain samples were obtained from the Human Brain Transcriptome project (https://www.hbatlas.org) (Kang et al., 2011). Heatmaps showing relative *BMP2*, *BMP4,* and *BMP7* expression by in situ hybridization (ISH) in murine brain across development were obtained from the ALLEN Developing Mouse Brain Atlas (https://www.developingmouse.brain-map.org; Lein et al., 2007).

#### scRNA-seq analyses of H3.3K27M ACVR1 WT DMG

Using the scRNA-seq data published by Jessa et al., 2022 (GSE210568) , we selected H3.3K27M-*ACVR1* WT pDMGs localized either in the pons or in the thalamus (10 samples). The same processing steps as the authors were performed except for the regression of mitochondrial proportion and the number of UMIs. Normalization was performed with the LogNormalize function of Seurat (Hao et al., 2021). All samples were filtered to keep tumoral cells as identified by the authors. As a readout of BMP pathway activity, we used PROGENy to infer the TGF-β/BMP score. We then stratified cells into TGF-β-High and TGF-β-Low groups using the 95^th^ quantile as the cutoff. FindMarkers with default parameters was used to identify differentially expressed (DE) genes (FDR ≤0.05) in each group. Enrichment analyzes were then conducted using the enrichR package (Kuleshov et al., 2016) and the Gene Ontology Biological Process gene signatures (Ashburner et al., 2000), separately on DE genes upregulated in TGF-β-High and TGF-β-Low for each sample. Only the top 10 significant enrichments (FDR ≤0.05) are shown on the plots. Identification of DE BMP ligands and receptors was run with FindMarkers using a logfc.threshold of 0.15 (instead of the default 0.25). For the visualization of all samples on a shared UMAP, Harmony (Korsunsky et al., 2019) (v 0.1.1) was used to integrate the 10 samples on the 50 principal components computed. The first 30 harmony-corrected principal components were then used to compute the SNN graph and UMAP.

### RNA-sequencing of in-house pDMG samples

As part of the Share4Kids program, a third cohort was constituted from leftover DMG samples, obtained through biopsies performed at the Pediatric Hematology and Oncology Institute (iHOPE, Lyon) and the Hôpital Femme Mère Enfant (HFME, Lyon). Tissue banking and research were conducted according to national ethics guidelines, after obtaining the written informed consent of patients. This study was approved by the ethical review board of the BRC of the Centre Léon Bérard (noBB-0033–00050, No 2020–02). This BRC quality is certified according to AFNOR NFS96900 (No 2009/35884.2) and ISO 9001 (Certification N° 2013/56348.2). Biological material collection and retention activity are declared to the Ministry of Research (DC-2008–99 and AC-2019–3426). For RNA-seq library construction of cohort 3, total RNAs from tissues were isolated using the AllPrep DNA/RNA FFPE kit (Qiagen, 80224) following the manufacturer’s instructions. Libraries were prepared with Illumina Stranded mRNA Prep (Illumina, 20040534) following recommendations. Quality was further assessed using the TapeStation 4200 automated electrophoresis system (Agilent) with High Sensitivity D1000 ScreenTape (Agilent). All libraries were sequenced (2×100 bp) using Agilent SureSelect RNA XTHS2 All Exon V8 (Agilent, G9991A) according to the standard Agilent protocol.

Quality control of reads was performed using FastQC (v.0.11.9) (Andrews, 2010), followed by trimming of Illumina adapter sequences with Cutadapt (Martin, 2011) (v.3.4) using the -a CTGTCTCTTATACACATCT and -A CTGTCTCTTATACACATCT parameters. Reads were mapped to the GRCh38 human genome using ‘two-pass’ mode STAR (v.2.7.9) (Dobin et al., 2013), with Ensembl v104 annotations. Gene counts were then computed using HTseq-count (v.0.13.5) (Anders et al., 2014) with the following parameters: ‘--order pos’ and ‘--stranded reverse.’ HTseq count files were then loaded in R (v 4.2.0) and two filtration steps were applied using the annotations of org.Hs.eg.db (v 3.15.0) Carlson et al., 2019: genes with low counts (less than 10 reads across samples) were removed; non-protein-coding genes were removed. Filtered gene counts were converted into a DESeq2 object with the design parameter set to account for the tumor histone mutation (HGG/DIPG) and normalized with vst using ‘blind = FALSE.’. The R package ggplot2 (v 3.3.5) (Villanueva and Chen, 2019) was used to plot the expression of BMP ligands.

### Spatial transcriptomics analyzes

FFPE tissue sections were placed on Visium slides and prepared according to 10X Genomics protocols. After H&E staining, imaging and decrosslinking steps, tissue sections were incubated with human-specific probes targeting 17,943 genes (10X genomics, Visium Human Transcriptome Probe Set v.1.0). Probes hybridised on mRNA were captured on Visium slides and a gene expression library prepared following 10X genomics dedicated protocol and sequenced on Illumina NovaSeq 6000 targeting 50,000 reads per spot.

Raw reads were pre-processed using the space ranger count pipeline (v 2.0.0) and the human GRCh38 reference provided by 10X Genomics. Filtered H5 matrices were loaded using the Load10X_Spatial function from Seurat. The following pipeline was applied to process raw counts: counts were normalized with the LogNormalise method and a scale factor of 10000; the top 4000 variable features (‘nfeatures’ parameter) were identified using the vst method; data were then scaled for all genes, the first 50 components of the PCA were computed using the 4000 variable features identified; SNN graph was constructed using the first 30 dimensions of the PCA (dims parameter); clusters were identified with the Louvain algorithm along several resolutions ranging from 0.1 to 1; UMAP was also computed using the first 30 dimensions of the PCA (dims parameter). Signatures identified by Ren et al., 2023 and Filbin et al., 2018 were used in conjunction with sc-type (Ianevski et al., 2022) to annotate the clusters at a resolution of 0.5. Annotated clusters were validated with an anatomopathologist, leading to the sub clusterization of the ‘Invasive niche’ of HFME-1 into 2 new clusters (‘Invasive_niche_1’ and ‘Invasive_niche_2’). Scoring of Ren’s signatures were made with the AddModuleScore function of Seurat. PROGENy was used to score TGF-β/BMP pathway activity. Markers of the ‘Invasive niche’ were identified with the FindMarkers function using a logfc.threshold=0.15.

### Cell culture and treatments

Human pediatric low-grade glioma cell line Res259 (grade II, diffuse astrocytoma) and high-grade glioma cell line SF188 (grade IV, glioblastoma) were kindly provided by Dr Samuel Meignan. Both isogenic cell lines overexpressing H3.3 wild-type and H3.3K27M were generated as previously described by Rakotomalala et al., 2021. All cell lines were grown as a monolayer in DMEM medium with GlutaMAX-I, 4,5 g/L D-Glucose and 110 mg/L pyruvate (Gibco, 31966) supplemented with 10% foetal bovine serum (FBS), 1 X MEM non-essential amino acid solution (Gibco, 11140050) and 1 X Penicillin-Streptomycin (Gibco, 15140122). Cells were incubated in a humid atmosphere at 37 °C with 5% CO2.

To assess the impact of BMP7 treatment on wild-type or H3.3K27M-mutated Res259 or SF188 cell lines, 1.5x10^5^ cells were plated into six-well plates in a complete medium. After 24 hr, cells were rinsed with PBS and fresh medium and serum-deprived in 1% FBS medium for 3 hr. 50 ng/mL of human recombinant BMP7 (Peprotech, 120–03 P) was then directly added to the medium, and cells were harvested at indicated time points for further experiments.

For pDMG cell lines grown as spheroids, HSJD-DIPG-007, HSJD-DIPG-012, HSJD-DIPG-013, and HSJD-DIPG-014 were kindly provided by Dr Angel Montero-Carcaboso. BT245 and SU-DIPGXIII, KO or not for H3.3K27M mutation, were kindly provided by Dr Nada Jabado (Harutyunyan et al., 2019). pDMG cell lines were grown in a complete culture medium as described by Monje et al., 2011, in 96-well ULA plates (Corning, 7007) or 25 cm² low attachment culture flasks (Corning, 431463). The Medium was changed twice a week and spheroids were splitted every 1–2 weeks when reaching 800–1000 µm of diameter using TrypLE Express Enzyme (Thermo Fisher Scientific, 12605010) preheated to 37 °C. Hypoxia and ONC201 treatments were induced on HSJD-DIPG-012 spheroids once a diameter of 600 µm was reached. To induce hypoxia, HSJD-DIPG-012 spheres were incubated for 3 hr in a dedicated incubator at 37 °C, 1% O_2_, while the controls were incubated at 37 °C in normoxia conditions. Spheroids were similarly treated or not with 20 µM of ONC201 (Selleckchem, S7963) for 96 hr. Exploration of BMP2 effect on HSJD-DIPG-012 and HSJD-DIPG-014 spheroids was done using 10 ng/mL of human recombinant BMP2 (Peprotech, 120–02 C) before protein quantification by western blot.

All cultures were tested every month for mycoplasma using the MycoAlert Mycoplasma Detection Kit (Lonza, LT07-318), in accordance with the manufacturer’s instructions.

### RNA-seq data processing and analyses of Res259 cells

For RNA-seq library construction, 1000 ng of total RNAs were isolated using the Nucleospin RNA kit (Macherey-Nagel, 740955) following the manufacturer’s instructions. Libraries were prepared with Illumina Stranded mRNA Prep (Illumina, 20040534) following recommendations. Quality was further assessed using the TapeStation 4200 automated electrophoresis system (Agilent) with High Sensitivity D1000 ScreenTape (Agilent). All libraries were sequenced (2×75 bp) using NovaSeq 6000 (Illumina) according to the standard Illumina protocol.

Raw sequence quality was assessed using FastQC (0.11.9) (Andrews, 2010). The trimming step was omitted, as 5’ and 3’ read bases had a quality greater than Q30, and no adapter fragments were detected. Reads were then pseudo-aligned using Kallisto (0.46.2) (Bray et al., 2016) with Ensembl v96, human genome build GRCh38. The rest of the analyzes were performed using R version (4.2.0). Differential expression (DE) analyzes were conducted using the DESeq2 package (1.36.0) (Love et al., 2014) using default parameters. Genes with corrected *p*-value (Benjamini–Hochberg)<0.05 and |log2FoldChange (LFC)|>log2(1.5) were considered DE. EnrichR package (3.0) (Kuleshov et al., 2016) was used for overrepresentation analysis using the following databases: Gene Ontology (GO) Biological Process 2021, GO Molecular Function 2021, GO Cellular Compartment 2021, KEGG 2021 Human, MSigDB Hallmarks 2020 and ChEA 2016. Enrichment significance was assessed by Fisher’s exact test, and *p*-values were corrected with the Bonjamini-Hochberg method. Overrepresentation analyses were run separately on upregulated and downregulated DE genes. PCA was performed using FactoMineR and plotted with factoextra. Boxplots and heatmaps were respectively made with ggplot2 and ComplexHeatmap (v 2.12.1) (Gu et al., 2016) using the vst normalized expression. TGF-β pathway activity was inferred with PROGENy using the first 100 genes of the model (top = 100).

### qRT-PCR profiling

Total RNA was extracted using a Nucleospin RNA kit (Macherey-Nagel, 740955) or RNeasy Plus Micro (Qiagen, 74034) following the manufacturer’s instructions. 500–1000 ng was reverse transcribed using the iScript cDNA Synthesis kit (Bio-Rad, 1708891) according to the manufacturer’s instructions. Expression of *BMP2* [forward primer (5′-TGCGGTCTCCTAAAGGTCG-3′) and reverse primer (5′-GAATTCAGAAGCCTGCAAGG-3′)], *BMP7* [forward primer (5′-GGGTGGGTCTCTGTTTCAG-3′) and reverse primer (5′-CCTGGAGCACCTGATAAACG-3′)], *ID1* [forward primer (5′-GGTGCGCTGTCTGTCTGAG-3′) and reverse primer (5′-TGTCGTAGAGCAGCACGTTT-3′)] and *ID2* [forward primer (5′-CCCAGAACAAGAAGGTGAGC-3′) and reverse primer (5′-GAATTCAGAAGCCTGCAAGG-3′)], were assessed by real-time quantitative QRT-PCR on a LightCycler 480 instrument (Roche) using the LightCycler 480 SYBR Green I Master Mix (Roche, 04707516001), according to the manufacturer’s instructions. *HPRT1* [forward primer (5′-AAGAGCTATTGTAATGACCAGT-3′) and reverse primer (5′-CAAAGTCTGCATTGTTTTGC-3′)] expression was used as a housekeeping gene.

The expression of a panel of 84 genes of the TGF-β/BMP signaling pathway was assessed in HSJD-DIPG-007, HSJD-DIPG-012, and HSJD-DIPG-014 cell lines by real-time quantitative QRT-PCR on a LightCycler 480 instrument (Roche) using an RT² Profiler PCR Array (Qiagen, 330231), according to the manufacturer’s instructions. RNA were extracted using the RNeasy mini kit (Qiagen, 74104), and 500 ng of RNA were reverse transcribed using the RT2 First Strand Kit (Qiagen, 330401) according to the manufacturer’s instructions.

### Western blot

Cells were lysed in RIPA Buffer (50 mM Tris-HCl pH 8, 150  mM NaCl, IGEPAL 1%, 0.5% sodium deoxycholate, 0.1% SDS) containing a protease and phosphatase inhibitor cocktail (Thermo Fisher Scientific, 78440). Protein contents were estimated using the Thermo Scientific Pierce BCA Protein Assay Kit (Fischer Scientific, 23225). Samples were diluted with distilled water to achieve equal concentrations and a loading buffer (4 x Laemmli Sample Buffer, Biorad, 1610747) containing 100 mM DTT (Sigma-Aldrich, 11583786001) was added. Protein extracts were then analyzed by immunoblot. Briefly, proteins were loaded into SDS‐polyacrylamide gels for electrophoresis (Mini Protean TGX gels, Biorad, 4561034) and blotted onto polyvinylidene fluoride sheets (Trans-Blot Turbo Transfer PVDF pack, Biorad, 1704156) using the TransBlot technology (Bio-Rad). Membranes were blocked with 5% BSA in TBS-Tween 0.1% for 1 hr and then incubated overnight at 4 °C with anti-pSMAD1/5/8 (1:1000, Cell Signaling Technology, 13820), anti-SMAD1 (1:1000, Cell Signaling Technology, 6944), anti-SMAD1/5/8 (1:1000, Abcam, ab80255), anti-pRb (1:500, Abcam, ab47763), and anti-Rb (1:1000, Cell Signaling Technology, 9309). After three washes with TBS-Tween 0.1%, membranes were incubated with the appropriate HRP-conjugated secondary antibody (1:20.000, Jackson ImmunoResearch). HRP-conjugated β-Actin antibody (1:25.000, Sigma-Aldrich A3854) and HRP-conjugated GAPDH antibody (1:2000 Cell Signaling Technology, 8884), used as loading controls, were incubated for 1  h at room temperature. After three washes with TBS-Tween 0.1%, the detection was performed using the Amersham ECL Prime Detection Reagent (Cytiva, RPN2232). Membranes were imaged on the ChemiDoc Touch Imaging System (Bio-Rad).

### Proliferation and cell cycle characterization assays

For proliferation assay, 5×10^4^ Res259 or 7.5x10^4^ SF188 cells were plated into six-well plates in a complete medium. After 72 hr of BMP7 treatment, total cell number and viability were quantified by image cytometry on a NucleoCounter NC-3000 (Chemometec) according to the procedure provided by the manufacturer, using a co-staining of Acridine Orange and DAPI (Chemometec, 910–3013).

For cell cycle analyses, 3×10^5^ (Hoffman et al., 2018) Res259 or SF188 cells were plated into 10 cm-petri dishes in a complete medium. After 24 hr of BMP7 treatment, 10 μM EdU was added for 1.5 hr. Cells were then harvested using Trypsin (Gibco, 25300054) and washed twice with PBS. Click-it reactions were performed using the Click-iT Plus EdU Alexa Fluor 647 Flow Cytometry Kit (Invitrogen, C10634), according to the manufacturer’s instructions. Cells were counterstained with DAPI and analyzed on a BD FACSAria Fusion flow cytometer (BD Biosciences). Data analysis was performed with FlowJo v10.7.1 Software (BD Biosciences). Cells were identified on a Side Scatter (SSC) vs Forward Scatter (FSC) dot plot and cell debris and aggregates were excluded from analysis based on FSC signals.

For β-galactosidase activity determination, 5×10^3^ Res259 cells were plated into 12-well plates in a complete medium. After 72 hr of BMP7 treatment, cells were fixed for 5 min in 0.5% glutaraldehyde, rinsed twice with PBS, and incubated for 48 hr at 37 °C in senescence-associated beta-galactosidase (SA-β-Gal) staining solution as previously described (Debacq-Chainiaux et al., 2009). SA-β-Gal + cells were counted manually, and a total number of cells were counted using a DAPI-counterstaining and using the Fiji software (Schindelin et al., 2012).

### Invasion assays

For 2D pediatric glioma cell lines (Res259 and SF188), 8.0 µm-Boyden chambers (Falcon, 353097) were coated with 100 µL of 10% Geltrex LDEV-Free, hESC-Qualified, Reduced Growth Factor Basement Membrane Matrix (Gibco, A1413301) completed or not with 50 ng/mL human recombinant BMP7. During the 3 days preceding the experiment, Res259 WT or K27M cells were pre-conditioned or not with 50 ng/mL human recombinant BMP7. 2.5×10^4^ cells were plated over Geltrex in 400 µL 1% FBS-DMEM completed or not with 50 ng/mL human recombinant BMP7. Boyden chambers were deposited in 24-well plates filled with 700 µL of 10% FBS-DMEM. After 48 hr, Boyden chambers were washed with PBS, and incubated for 20 min with methanol to fix the cells, and Crystal violet 0,1% (Sigma-Aldrich, V5265) was used to color cells allowing counting. Pictures of the chambers were taken using an EVOS-7000 (Invitrogen) and analyzed using the Fiji software.

For pDMG cell lines grown as spheroids, HSJD-DIPG-012 were plated in 10% Matrigel (Corning, 354277) in Nunc Lab-Tek Chamber Slide (Thermo Fisher Scientific, 177402). Media and matrigel were supplemented with either 10 ng/mL human recombinant BMP2 or 1 µM LDN-193189 (Selleckchem, S2618). Invasiveness was measured at 48 hr after seeding. Images were captured using an EVOS-7000 (Invitrogen) and analyzed using the ‘ROI’ feature in the Fiji software.

### Spheroids growth monitoring

To assess the effects of BMP2 on pDMG spheroids, 1×10^4^ cells of HSJD-DIPG-012 or HSJD-DIPG-014 were seeded in 96-well plates (Corning, 7007). Cells were treated at seeding with concentrations of 1, 5, 20, 50, or 200 ng/mL of human recombinant BMP2 (Peprotech, 120–02 C). Media were renewed twice a week, with or without recombinant BMP2. Cell growth was monitored using an EVOS-7000 (Invitrogen) and pictures of control and treated 6 days or 9 days spheres were used to calculate sphere area of HSJD-DIPG-014 and HSJD-DIPG-012, respectively.

### Immunohistochemistry

HSJD-DIPG-012 spheroids were treated when reaching a diameter of 600 µm either with 200 ng/mL of human recombinant BMP2 or with 1 µM of LDN-193189. Spheroids were then fixed in 4% PFA for 1 hr at room temperature, dehydrated, and embedded in paraffin. 8 µm-sections were then deparaffinized, rehydrated, and heated in a citrate buffer (0.01 M; pH 6.0) for 15 min. Sections were then incubated overnight at 4 °C with the appropriate dilution of anti-Ki67 (1:100, Dako, M7240) in TBS containing Horse Serum (2%). After several washes in TBS-Tween 0.05 %, sections were incubated with the secondary antibody for 1 hr, and then washed again in TBS-Tween 0.05%. Color was developed with 3,3’-diaminobenzidine (DAB, Vector Laboratories, SK-4100) incubation for 1–3 min and with Hematoxylin (Vector Laboratories, H-3401) incubation for 1 min. Images were captured using an EVOS-7000 (Invitrogen) and quantification of Ki67-positive cells was assessed using the ‘Cell Positive Detection’ QuPath function Bankhead et al., 2017, with parameters: ‘Optical density sum,’ ‘thresholdCompartment:’ ‘Cell: DAB OD mean,’ ‘thresholdPositive2:’ 0.25.

### Statistical analyses

Data are represented as means ± std. Sample size and replicates are stated in the corresponding figure legends. Using GraphPad Prism 9 software and R (4.2.0), the statistical significance between the two groups were determined by one-tailed Mann-Whitney signed-rank tests, apart from the proportion of Ki67 stained cells, which was assessed using Fisher’s exact Test. The following symbols used to denote (<0.05: *;<0.01: **;<0.001: ***;<0.0001: ****).

## Data Availability

Publicly available RNA-seq data of pDMG samples from cohort 1 were obtained from St. Jude Cloud (McLeod et al., 2021; https://platform.stjude.cloud/data/diseases/tumor) upon DTA. Cohort 2 of glioma samples from Mackay et al., 2017 can be accessed on https://pedcbioportal.kidsfirstdrc.org/ with the following dataset id: “phgg_jones_meta_2017”. The scRNA-seq cohort of H3.3K27M ACVR1 WT pDMGs is available on GEO (GSE210568) and Zenodo. Share4Kids transcriptomic data from cohort 3 are described in Supplementary file 1c and d. They are available upon request via the Share4Kids data portal. Users must request access by emailing thomas.diot@lyon.unicancer.fr with a project summary (to comply with the RGPD framework), and access will be automatically granted without any assessment other than checking the ethics of the objectives. They will then have access to a space enabling them to interrogate the data with dedicated analysis tools. RNAseq on glioma cell lines exposed or not to BMP7 are available on GEO (GSE268576). Spatial transcriptomic data on patient samples can be similarly accessed on GEO (GSE268577). The code used to generate the figures has been made publicly available on GitLab under an open source license (copy archived at Meyer, 2024). The following datasets were generated: HuchedéP MeyerS MeignanS CastetsM 2023Transcriptomic modifications induced by BMP depend on H3.3K27M contextNCBI Gene Expression OmnibusGSE268576 HuchedéP MeyerS DegletagneC VasiljevicA CastetsM 2023Spatial transcriptomic analyses of DIPG samplesNCBI Gene Expression OmnibusGSE268577 The following previously published datasets were used: JessaS HebertS KleinmanCL 2022HGG-oncohistones processed dataNCBI Gene Expression OmnibusGSE210568 JessaS HébertS KleinmanCL 2022HGG-oncohistones processed dataZenodo10.5281/zenodo.6929428

## Additional files

Supplementary file 1. Analysis of the profile of expression of BMP pathway in pDMG. (**a**) List of the BMP target genes used for the heatmaps shown in Figure 1B and Figure 1—figure supplement 1B. (**b**) Average BMP ligand expression (natural log) per sample in the spatial transcriptomic dataset of DMG samples. (**c**) Normalized expression matrix (vst) of BMP pathway genes from cohort 3, including those shown in Figure 1—figure supplement 1E. (**d**) Histone mutation of samples from cohort 3.

MDAR checklist

## References

[bib1] Afrakhte M, Morén A, Jossan S, Itoh S, Sampath K, Westermark B, Heldin CH, Heldin NE, ten Dijke P (1998). Induction of inhibitory Smad6 and Smad7 mRNA by TGF-beta family members. Biochemical and Biophysical Research Communications.

[bib2] Akizu N, Estarás C, Guerrero L, Martí E, Martínez-Balbás MA (2010). H3K27me3 regulates BMP activity in developing spinal cord. Development.

[bib3] Anders S, Huber W (2010). Differential expression analysis for sequence count data. Genome Biology.

[bib4] Anders S, Pyl PT, Huber W (2014). A python framework to work with high-throughput sequencing data. bioRxiv.

[bib5] Andrews S (2010). FastQC: a quality control tool for high throughput sequence data.

[bib6] Antonica F, Santomaso L, Pernici D, Petrucci L, Aiello G, Cutarelli A, Conti L, Romanel A, Miele E, Tebaldi T, Tiberi L (2022). A slow-cycling/quiescent cells subpopulation is involved in glioma invasiveness. Nature Communications.

[bib7] Ashburner M, Ball CA, Blake JA, Botstein D, Butler H, Cherry JM, Davis AP, Dolinski K, Dwight SS, Eppig JT, Harris MA, Hill DP, Issel-Tarver L, Kasarskis A, Lewis S, Matese JC, Richardson JE, Ringwald M, Rubin GM, Sherlock G (2000). Gene Ontology: tool for the unification of biology. Nature Genetics.

[bib8] Atkins RJ, Stylli SS, Kurganovs N, Mangiola S, Nowell CJ, Ware TM, Corcoran NM, Brown DV, Kaye AH, Morokoff A, Luwor RB, Hovens CM, Mantamadiotis T (2019). Cell quiescence correlates with enhanced glioblastoma cell invasion and cytotoxic resistance. Experimental Cell Research.

[bib9] Bankhead P, Loughrey MB, Fernández JA, Dombrowski Y, McArt DG, Dunne PD, McQuaid S, Gray RT, Murray LJ, Coleman HG, James JA, Salto-Tellez M, Hamilton PW (2017). QuPath: Open source software for digital pathology image analysis. Scientific Reports.

[bib10] Bénazet J-D, Bischofberger M, Tiecke E, Gonçalves A, Martin JF, Zuniga A, Naef F, Zeller R (2009). A self-regulatory system of interlinked signaling feedback loops controls mouse limb patterning. Science.

[bib11] Bender S, Tang Y, Lindroth AM, Hovestadt V, Jones DTW, Kool M, Zapatka M, Northcott PA, Sturm D, Wang W, Radlwimmer B, Højfeldt JW, Truffaux N, Castel D, Schubert S, Ryzhova M, Seker-Cin H, Gronych J, Johann PD, Stark S, Meyer J, Milde T, Schuhmann M, Ebinger M, Monoranu C-M, Ponnuswami A, Chen S, Jones C, Witt O, Collins VP, von Deimling A, Jabado N, Puget S, Grill J, Helin K, Korshunov A, Lichter P, Monje M, Plass C, Cho Y-J, Pfister SM (2013). Reduced H3K27me3 and DNA hypomethylation are major drivers of gene expression in K27M mutant pediatric high-grade gliomas. Cancer Cell.

[bib12] Blanco-Suarez E, Liu TF, Kopelevich A, Allen NJ (2018). Astrocyte-secreted chordin-like 1 drives synapse maturation and limits plasticity by increasing synaptic GluA2 AMPA Receptors. Neuron.

[bib13] Bray NL, Pimentel H, Melsted P, Pachter L (2016). Near-optimal probabilistic RNA-seq quantification. Nature Biotechnology.

[bib14] Buczkowicz P, Hoeman C, Rakopoulos P, Pajovic S, Letourneau L, Dzamba M, Morrison A, Lewis P, Bouffet E, Bartels U, Zuccaro J, Agnihotri S, Ryall S, Barszczyk M, Chornenkyy Y, Bourgey M, Bourque G, Montpetit A, Cordero F, Castelo-Branco P, Mangerel J, Tabori U, Ho KC, Huang A, Taylor KR, Mackay A, Bendel AE, Nazarian J, Fangusaro JR, Karajannis MA, Zagzag D, Foreman NK, Donson A, Hegert JV, Smith A, Chan J, Lafay-Cousin L, Dunn S, Hukin J, Dunham C, Scheinemann K, Michaud J, Zelcer S, Ramsay D, Cain J, Brennan C, Souweidane MM, Jones C, Allis CD, Brudno M, Becher O, Hawkins C (2014). Genomic analysis of diffuse intrinsic pontine gliomas identifies three molecular subgroups and recurrent activating ACVR1 mutations. Nature Genetics.

[bib15] Burford A (2021). Of mice and men: translating mouse age to human age.

[bib16] Carlson M, Falcon S, Pages H, Li N (2019). R Package.

[bib17] Carvalho D, Taylor KR, Olaciregui NG, Molinari V, Clarke M, Mackay A, Ruddle R, Henley A, Valenti M, Hayes A, Brandon ADH, Eccles SA, Raynaud F, Boudhar A, Monje M, Popov S, Moore AS, Mora J, Cruz O, Vinci M, Brennan PE, Bullock AN, Carcaboso AM, Jones C (2019). ALK2 inhibitors display beneficial effects in preclinical models of *ACVR1* mutant diffuse intrinsic pontine glioma. Communications Biology.

[bib18] Chan K-M, Fang D, Gan H, Hashizume R, Yu C, Schroeder M, Gupta N, Mueller S, James CD, Jenkins R, Sarkaria J, Zhang Z (2013). The histone H3.3K27M mutation in pediatric glioma reprograms H3K27 methylation and gene expression. Genes & Development.

[bib19] Chen J, Li Y, Yu T-S, McKay RM, Burns DK, Kernie SG, Parada LF (2012). A restricted cell population propagates glioblastoma growth after chemotherapy. Nature.

[bib20] Christiaen L, Stolfi A, Levine M (2010). BMP signaling coordinates gene expression and cell migration during precardiac mesoderm development. Developmental Biology.

[bib21] Debacq-Chainiaux F, Erusalimsky JD, Campisi J, Toussaint O (2009). Protocols to detect senescence-associated beta-galactosidase (SA-betagal) activity, a biomarker of senescent cells in culture and in vivo. Nature Protocols.

[bib22] Di Ruscio V, Del Baldo G, Fabozzi F, Vinci M, Cacchione A, de Billy E, Megaro G, Carai A, Mastronuzzi A (2022). Pediatric diffuse midline gliomas: an unfinished puzzle. Diagnostics.

[bib23] Dobin A, Davis CA, Schlesinger F, Drenkow J, Zaleski C, Jha S, Batut P, Chaisson M, Gingeras TR (2013). STAR: ultrafast universal RNA-seq aligner. Bioinformatics.

[bib24] Filbin MG, Tirosh I, Hovestadt V, Shaw ML, Escalante LE, Mathewson ND, Neftel C, Frank N, Pelton K, Hebert CM, Haberler C, Yizhak K, Gojo J, Egervari K, Mount C, van Galen P, Bonal DM, Nguyen Q-D, Beck A, Sinai C, Czech T, Dorfer C, Goumnerova L, Lavarino C, Carcaboso AM, Mora J, Mylvaganam R, Luo CC, Peyrl A, Popović M, Azizi A, Batchelor TT, Frosch MP, Martinez-Lage M, Kieran MW, Bandopadhayay P, Beroukhim R, Fritsch G, Getz G, Rozenblatt-Rosen O, Wucherpfennig KW, Louis DN, Monje M, Slavc I, Ligon KL, Golub TR, Regev A, Bernstein BE, Suvà ML (2018). Developmental and oncogenic programs in H3K27M gliomas dissected by single-cell RNA-seq. Science.

[bib25] Fontebasso AM, Papillon-Cavanagh S, Schwartzentruber J, Nikbakht H, Gerges N, Fiset P-O, Bechet D, Faury D, De Jay N, Ramkissoon LA, Corcoran A, Jones DTW, Sturm D, Johann P, Tomita T, Goldman S, Nagib M, Bendel A, Goumnerova L, Bowers DC, Leonard JR, Rubin JB, Alden T, Browd S, Geyer JR, Leary S, Jallo G, Cohen K, Gupta N, Prados MD, Carret A-S, Ellezam B, Crevier L, Klekner A, Bognar L, Hauser P, Garami M, Myseros J, Dong Z, Siegel PM, Malkin H, Ligon AH, Albrecht S, Pfister SM, Ligon KL, Majewski J, Jabado N, Kieran MW (2014). Recurrent somatic mutations in ACVR1 in pediatric midline high-grade astrocytoma. Nature Genetics.

[bib26] Fortin J, Tian R, Zarrabi I, Hill G, Williams E, Sanchez-Duffhues G, Thorikay M, Ramachandran P, Siddaway R, Wong JF, Wu A, Apuzzo LN, Haight J, You-Ten A, Snow BE, Wakeham A, Goldhamer DJ, Schramek D, Bullock AN, Dijke PT, Hawkins C, Mak TW (2020). Mutant ACVR1 arrests glial cell differentiation to drive tumorigenesis in pediatric gliomas. Cancer Cell.

[bib27] Furst L, Atkins RJ, Dinevska M, Stylli SS, Corcoran NM, Hovens CM, Mantamadiotis T (2022). Identification and isolation of slow-cycling glioma stem cells. Methods in Cell Biology.

[bib28] Gu Z, Eils R, Schlesner M (2016). Complex heatmaps reveal patterns and correlations in multidimensional genomic data. Bioinformatics.

[bib29] Hao Y, Hao S, Andersen-Nissen E, Mauck WM, Zheng S, Butler A, Lee MJ, Wilk AJ, Darby C, Zager M, Hoffman P, Stoeckius M, Papalexi E, Mimitou EP, Jain J, Srivastava A, Stuart T, Fleming LM, Yeung B, Rogers AJ, McElrath JM, Blish CA, Gottardo R, Smibert P, Satija R (2021). Integrated analysis of multimodal single-cell data. Cell.

[bib30] Harutyunyan AS, Krug B, Chen H, Papillon-Cavanagh S, Zeinieh M, De Jay N, Deshmukh S, Chen CCL, Belle J, Mikael LG, Marchione DM, Li R, Nikbakht H, Hu B, Cagnone G, Cheung WA, Mohammadnia A, Bechet D, Faury D, McConechy MK, Pathania M, Jain SU, Ellezam B, Weil AG, Montpetit A, Salomoni P, Pastinen T, Lu C, Lewis PW, Garcia BA, Kleinman CL, Jabado N, Majewski J (2019). H3K27M induces defective chromatin spread of PRC2-mediated repressive H3K27me2/me3 and is essential for glioma tumorigenesis. Nature Communications.

[bib31] Hatsell SJ, Idone V, Wolken DMA, Huang L, Kim HJ, Wang L, Wen X, Nannuru KC, Jimenez J, Xie L, Das N, Makhoul G, Chernomorsky R, D’Ambrosio D, Corpina RA, Schoenherr CJ, Feeley K, Yu PB, Yancopoulos GD, Murphy AJ, Economides AN (2015). ACVR1R206H receptor mutation causes fibrodysplasia ossificans progressiva by imparting responsiveness to activin A. Science Translational Medicine.

[bib32] Haupt J, Xu M, Shore EM (2018). Variable signaling activity by FOP ACVR1 mutations. Bone.

[bib33] Hawley SH, Wünnenberg-Stapleton K, Hashimoto C, Laurent MN, Watabe T, Blumberg BW, Cho KW (1995). Disruption of BMP signals in embryonic *Xenopus* ectoderm leads to direct neural induction. Genes & Development.

[bib34] Hino K, Ikeya M, Horigome K, Matsumoto Y, Ebise H, Nishio M, Sekiguchi K, Shibata M, Nagata S, Matsuda S, Toguchida J (2015). Neofunction of ACVR1 in fibrodysplasia ossificans progressiva. PNAS.

[bib35] Hoeman CM, Cordero FJ, Hu G, Misuraca K, Romero MM, Cardona HJ, Nazarian J, Hashizume R, McLendon R, Yu P, Procissi D, Gadd S, Becher OJ (2019). ACVR1 R206H cooperates with H3.1K27M in promoting diffuse intrinsic pontine glioma pathogenesis. Nature Communications.

[bib36] Hoffman LM, Veldhuijzen van Zanten SEM, Colditz N, Baugh J, Chaney B, Hoffmann M, Lane A, Fuller C, Miles L, Hawkins C, Bartels U, Bouffet E, Goldman S, Leary S, Foreman NK, Packer R, Warren KE, Broniscer A, Kieran MW, Minturn J, Comito M, Broxson E, Shih C-S, Khatua S, Chintagumpala M, Carret AS, Escorza NY, Hassall T, Ziegler DS, Gottardo N, Dholaria H, Doughman R, Benesch M, Drissi R, Nazarian J, Jabado N, Boddaert N, Varlet P, Giraud G, Castel D, Puget S, Jones C, Hulleman E, Modena P, Giagnacovo M, Antonelli M, Pietsch T, Gielen GH, Jones DTW, Sturm D, Pfister SM, Gerber NU, Grotzer MA, Pfaff E, von Bueren AO, Hargrave D, Solanki GA, Jadrijevic Cvrlje F, Kaspers GJL, Vandertop WP, Grill J, Bailey S, Biassoni V, Massimino M, Calmon R, Sanchez E, Bison B, Warmuth-Metz M, Leach J, Jones B, van Vuurden DG, Kramm CM, Fouladi M (2018). Clinical, radiologic, pathologic, and molecular characteristics of long-term survivors of diffuse intrinsic pontine glioma (DIPG): a collaborative report from the international and european society for pediatric oncology DIPG registries. Journal of Clinical Oncology.

[bib37] Huchedé P, Leblond P, Castets M (2022). The intricate epigenetic and transcriptional alterations in pediatric high-grade gliomas: targeting the crosstalk as the oncogenic achilles’ heel. Biomedicines.

[bib38] Ianevski A, Giri AK, Aittokallio T (2022). Fully-automated and ultra-fast cell-type identification using specific marker combinations from single-cell transcriptomic data. Nature Communications.

[bib39] Jackson ER, Duchatel RJ, Staudt DE, Persson ML, Mannan A, Yadavilli S, Parackal S, Game S, Chong WC, Jayasekara WSN, Le Grand M, Kearney PS, Douglas AM, Findlay IJ, Germon ZP, McEwen HP, Beitaki TS, Patabendige A, Skerrett-Byrne DA, Nixon B, Smith ND, Day B, Manoharan N, Nagabushan S, Hansford JR, Govender D, McCowage GB, Firestein R, Howlett M, Endersby R, Gottardo NG, Alvaro F, Waszak SM, Larsen MR, Colino-Sanguino Y, Valdés-Mora F, Rakotomalala A, Meignan S, Pasquier E, Andre N, Hulleman E, Eisenstat DD, Vitanza NA, Nazarian J, Koschmann C, Mueller S, Cain JE, Dun MD (2023). ONC201 in combination with paxalisib for the treatment of H3K27-altered diffuse midline glioma. Cancer Research.

[bib40] Jessa S, Mohammadnia A, Harutyunyan AS, Hulswit M, Varadharajan S, Lakkis H, Kabir N, Bashardanesh Z, Hébert S, Faury D, Vladoiu MC, Worme S, Coutelier M, Krug B, Faria Andrade A, Pathania M, Bajic A, Weil AG, Ellezam B, Atkinson J, Dudley RWR, Farmer J-P, Perreault S, Garcia BA, Larouche V, Blanchette M, Garzia L, Bhaduri A, Ligon KL, Bandopadhayay P, Taylor MD, Mack SC, Jabado N, Kleinman CL (2022). K27M in canonical and noncanonical H3 variants occurs in distinct oligodendroglial cell lineages in brain midline gliomas. Nature Genetics.

[bib41] Kang HJ, Kawasawa YI, Cheng F, Zhu Y, Xu X, Li M, Sousa AMM, Pletikos M, Meyer KA, Sedmak G, Guennel T, Shin Y, Johnson MB, Krsnik Z, Mayer S, Fertuzinhos S, Umlauf S, Lisgo SN, Vortmeyer A, Weinberger DR, Mane S, Hyde TM, Huttner A, Reimers M, Kleinman JE, Sestan N (2011). Spatio-temporal transcriptome of the human brain. Nature.

[bib42] Kassambara A, Mundt F (2017). CRAN.

[bib43] Khuong-Quang D-A, Buczkowicz P, Rakopoulos P, Liu X-Y, Fontebasso AM, Bouffet E, Bartels U, Albrecht S, Schwartzentruber J, Letourneau L, Bourgey M, Bourque G, Montpetit A, Bourret G, Lepage P, Fleming A, Lichter P, Kool M, von Deimling A, Sturm D, Korshunov A, Faury D, Jones DT, Majewski J, Pfister SM, Jabado N, Hawkins C (2012). K27M mutation in histone H3.3 defines clinically and biologically distinct subgroups of pediatric diffuse intrinsic pontine gliomas. Acta Neuropathologica.

[bib44] Kim HS, Neugebauer J, McKnite A, Tilak A, Christian JL (2019). BMP7 functions predominantly as a heterodimer with BMP2 or BMP4 during mammalian embryogenesis. eLife.

[bib45] Kluiver TA, Alieva M, van Vuurden DG, Wehrens EJ, Rios AC (2020). *Invaders Exposed:* understanding and targeting tumor cell invasion in diffuse intrinsic pontine glioma. Frontiers in Oncology.

[bib46] Korsunsky I, Millard N, Fan J, Slowikowski K, Zhang F, Wei K, Baglaenko Y, Brenner M, Loh P-R, Raychaudhuri S (2019). Fast, sensitive and accurate integration of single-cell data with Harmony. Nature Methods.

[bib47] Kozmikova I, Candiani S, Fabian P, Gurska D, Kozmik Z (2013). Essential role of Bmp signaling and its positive feedback loop in the early cell fate evolution of chordates. Developmental Biology.

[bib48] Kuleshov MV, Jones MR, Rouillard AD, Fernandez NF, Duan Q, Wang Z, Koplev S, Jenkins SL, Jagodnik KM, Lachmann A, McDermott MG, Monteiro CD, Gundersen GW, Ma’ayan A (2016). Enrichr: a comprehensive gene set enrichment analysis web server 2016 update. Nucleic Acids Research.

[bib49] Larman BW, Karolak MJ, Adams DC, Oxburgh L (2009). Chordin-like 1 and twisted gastrulation 1 regulate BMP signaling following kidney injury. Journal of the American Society of Nephrology.

[bib50] Lê S, Josse J, Husson F (2008). FactoMineR: an r package for multivariate analysis. Journal of Statistical Software.

[bib51] Lein ES, Hawrylycz MJ, Ao N, Ayres M, Bensinger A, Bernard A, Boe AF, Boguski MS, Brockway KS, Byrnes EJ, Chen L, Chen L, Chen T-M, Chin MC, Chong J, Crook BE, Czaplinska A, Dang CN, Datta S, Dee NR, Desaki AL, Desta T, Diep E, Dolbeare TA, Donelan MJ, Dong H-W, Dougherty JG, Duncan BJ, Ebbert AJ, Eichele G, Estin LK, Faber C, Facer BA, Fields R, Fischer SR, Fliss TP, Frensley C, Gates SN, Glattfelder KJ, Halverson KR, Hart MR, Hohmann JG, Howell MP, Jeung DP, Johnson RA, Karr PT, Kawal R, Kidney JM, Knapik RH, Kuan CL, Lake JH, Laramee AR, Larsen KD, Lau C, Lemon TA, Liang AJ, Liu Y, Luong LT, Michaels J, Morgan JJ, Morgan RJ, Mortrud MT, Mosqueda NF, Ng LL, Ng R, Orta GJ, Overly CC, Pak TH, Parry SE, Pathak SD, Pearson OC, Puchalski RB, Riley ZL, Rockett HR, Rowland SA, Royall JJ, Ruiz MJ, Sarno NR, Schaffnit K, Shapovalova NV, Sivisay T, Slaughterbeck CR, Smith SC, Smith KA, Smith BI, Sodt AJ, Stewart NN, Stumpf K-R, Sunkin SM, Sutram M, Tam A, Teemer CD, Thaller C, Thompson CL, Varnam LR, Visel A, Whitlock RM, Wohnoutka PE, Wolkey CK, Wong VY, Wood M, Yaylaoglu MB, Young RC, Youngstrom BL, Yuan XF, Zhang B, Zwingman TA, Jones AR (2007). Genome-wide atlas of gene expression in the adult mouse brain. Nature.

[bib52] Lewis PW, Müller MM, Koletsky MS, Cordero F, Lin S, Banaszynski LA, Garcia BA, Muir TW, Becher OJ, Allis CD (2013). Inhibition of PRC2 activity by a gain-of-function H3 mutation found in pediatric glioblastoma. Science.

[bib53] Li H (2011). Tabix: fast retrieval of sequence features from generic TAB-delimited files. Bioinformatics.

[bib54] Liem KF, Tremml G, Roelink H, Jessell TM (1995). Dorsal differentiation of neural plate cells induced by BMP-mediated signals from epidermal ectoderm. Cell.

[bib55] Lin J, Patel SR, Cheng X, Cho EA, Levitan I, Ullenbruch M, Phan SH, Park JM, Dressler GR (2005). Kielin/chordin-like protein, a novel enhancer of BMP signaling, attenuates renal fibrotic disease. Nature Medicine.

[bib56] Love MI, Huber W, Anders S (2014). Moderated estimation of fold change and dispersion for RNA-seq data with DESeq2. Genome Biology.

[bib57] Mackay A, Burford A, Carvalho D, Izquierdo E, Fazal-Salom J, Taylor KR, Bjerke L, Clarke M, Vinci M, Nandhabalan M, Temelso S, Popov S, Molinari V, Raman P, Waanders AJ, Han HJ, Gupta S, Marshall L, Zacharoulis S, Vaidya S, Mandeville HC, Bridges LR, Martin AJ, Al-Sarraj S, Chandler C, Ng H-K, Li X, Mu K, Trabelsi S, Brahim DH-B, Kisljakov AN, Konovalov DM, Moore AS, Carcaboso AM, Sunol M, de Torres C, Cruz O, Mora J, Shats LI, Stavale JN, Bidinotto LT, Reis RM, Entz-Werle N, Farrell M, Cryan J, Crimmins D, Caird J, Pears J, Monje M, Debily M-A, Castel D, Grill J, Hawkins C, Nikbakht H, Jabado N, Baker SJ, Pfister SM, Jones DTW, Fouladi M, von Bueren AO, Baudis M, Resnick A, Jones C (2017). Integrated molecular meta-analysis of 1,000 pediatric high-grade and diffuse intrinsic pontine glioma. Cancer Cell.

[bib58] Martin M (2011). Cutadapt removes adapter sequences from high-throughput sequencing reads. EMBnet.Journal.

[bib59] McLeod C, Gout AM, Zhou X, Thrasher A, Rahbarinia D, Brady SW, Macias M, Birch K, Finkelstein D, Sunny J, Mudunuri R, Orr BA, Treadway M, Davidson B, Ard TK, Chiao A, Swistak A, Wiggins S, Foy S, Wang J, Sioson E, Wang S, Michael JR, Liu Y, Ma X, Patel A, Edmonson MN, Wilkinson MR, Frantz AM, Chang T-C, Tian L, Lei S, Islam SMA, Meyer C, Thangaraj N, Tater P, Kandali V, Ma S, Nguyen T, Serang O, McGuire I, Robison N, Gentry D, Tang X, Palmer LE, Wu G, Suh E, Tanner L, McMurry J, Lear M, Pappo AS, Wang Z, Wilson CL, Cheng Y, Meshinchi S, Alexandrov LB, Weiss MJ, Armstrong GT, Robison LL, Yasui Y, Nichols KE, Ellison DW, Bangur C, Mullighan CG, Baker SJ, Dyer MA, Miller G, Newman S, Rusch M, Daly R, Perry K, Downing JR, Zhang J (2021). St. Jude cloud: a pediatric cancer genomic data-sharing ecosystem. Cancer Discovery.

[bib60] Messinger D, Harris MK, Cummings JR, Thomas C, Yang T, Sweha SR, Woo R, Siddaway R, Burkert M, Stallard S, Qin T, Mullan B, Siada R, Ravindran R, Niculcea M, Dowling AR, Bradin J, Ginn KF, Gener MAH, Dorris K, Vitanza NA, Schmidt SV, Spitzer J, Li J, Filbin MG, Cao X, Castro MG, Lowenstein PR, Mody R, Chinnaiyan A, Desprez P-Y, McAllister S, Dun MD, Hawkins C, Waszak SM, Venneti S, Koschmann C, Yadav VN (2023). Therapeutic targeting of prenatal pontine ID1 signaling in diffuse midline glioma. Neuro-Oncology.

[bib61] Meyer S (2024). Software Heritage.

[bib62] Monje M, Mitra SS, Freret ME, Raveh TB, Kim J, Masek M, Attema JL, Li G, Haddix T, Edwards MSB, Fisher PG, Weissman IL, Rowitch DH, Vogel H, Wong AJ, Beachy PA (2011). Hedgehog-responsive candidate cell of origin for diffuse intrinsic pontine glioma. PNAS.

[bib63] Mucha BE, Hashiguchi M, Zinski J, Shore EM, Mullins MC (2018). Variant BMP receptor mutations causing fibrodysplasia ossificans progressiva (FOP) in humans show BMP ligand-independent receptor activation in zebrafish. Bone.

[bib64] Nagaraja S, Quezada MA, Gillespie SM, Arzt M, Lennon JJ, Woo PJ, Hovestadt V, Kambhampati M, Filbin MG, Suva ML, Nazarian J, Monje M (2019). Histone variant and cell context determine H3K27M reprogramming of the enhancer landscape and oncogenic state. Molecular Cell.

[bib65] Nakayama N, Han CE, Scully S, Nishinakamura R, He C, Zeni L, Yamane H, Chang D, Yu D, Yokota T, Wen D (2001). A novel chordin-like protein inhibitor for bone morphogenetic proteins expressed preferentially in mesenchymal cell lineages. Developmental Biology.

[bib66] Piunti A, Hashizume R, Morgan MA, Bartom ET, Horbinski CM, Marshall SA, Rendleman EJ, Ma Q, Takahashi Y-H, Woodfin AR, Misharin AV, Abshiru NA, Lulla RR, Saratsis AM, Kelleher NL, James CD, Shilatifard A (2017). Therapeutic targeting of polycomb and BET bromodomain proteins in diffuse intrinsic pontine gliomas. Nature Medicine.

[bib67] Przystal JM, Cianciolo Cosentino C, Yadavilli S, Zhang J, Laternser S, Bonner ER, Prasad R, Dawood AA, Lobeto N, Chin Chong W, Biery MC, Myers C, Olson JM, Panditharatna E, Kritzer B, Mourabit S, Vitanza NA, Filbin MG, de Iuliis GN, Dun MD, Koschmann C, Cain JE, Grotzer MA, Waszak SM, Mueller S, Nazarian J (2022). Imipridones affect tumor bioenergetics and promote cell lineage differentiation in diffuse midline gliomas. Neuro-Oncology.

[bib68] Rakotomalala A, Bailleul Q, Savary C, Arcicasa M, Hamadou M, Huchedé P, Hochart A, Restouin A, Castellano R, Collette Y, Dieny E, Vincent A, Angrand P-O, Le Bourhis X, Leblond P, Furlan A, Castets M, Pasquier E, Meignan S (2021). H3.3K27M mutation controls cell growth and resistance to therapies in pediatric glioma cell lines. Cancers.

[bib69] Ramachandran A, Mehić M, Wasim L, Malinova D, Gori I, Blaszczyk BK, Carvalho DM, Shore EM, Jones C, Hyvönen M, Tolar P, Hill CS (2021). Pathogenic ACVR1^R206H^ activation by Activin A-induced receptor clustering and autophosphorylation. The EMBO Journal.

[bib70] Ren Y, Huang Z, Zhou L, Xiao P, Song J, He P, Xie C, Zhou R, Li M, Dong X, Mao Q, You C, Xu J, Liu Y, Lan Z, Zhang T, Gan Q, Yang Y, Chen T, Huang B, Yang X, Xiao A, Ou Y, Su Z, Chen L, Zhang Y, Ju Y, Zhang Y, Wang Y (2023). Spatial transcriptomics reveals niche-specific enrichment and vulnerabilities of radial glial stem-like cells in malignant gliomas. Nature Communications.

[bib71] Sakuta H, Suzuki R, Takahashi H, Kato A, Shintani T, Yamamoto TS, Ueno N, Noda M (2001). Ventroptin: a BMP-4 antagonist expressed in a double-gradient pattern in the retina. Science.

[bib72] Sánchez-de-Diego C, Valer JA, Pimenta-Lopes C, Rosa JL, Ventura F (2019). Interplay between BMPs and reactive oxygen species in cell signaling and pathology. Biomolecules.

[bib73] Schindelin J, Arganda-Carreras I, Frise E, Kaynig V, Longair M, Pietzsch T, Preibisch S, Rueden C, Saalfeld S, Schmid B, Tinevez J-Y, White DJ, Hartenstein V, Eliceiri K, Tomancak P, Cardona A (2012). Fiji: an open-source platform for biological-image analysis. Nature Methods.

[bib74] Schmid B (2000). Equivalent genetic roles for bmp7/snailhouse and bmp2b/swirl in dorsoventral pattern formation. Dev. Camb. Engl.

[bib75] Schubert M, Klinger B, Klünemann M, Sieber A, Uhlitz F, Sauer S, Garnett MJ, Blüthgen N, Saez-Rodriguez J (2018). Perturbation-response genes reveal signaling footprints in cancer gene expression. Nature Communications.

[bib76] Schwartzentruber J, Korshunov A, Liu X-Y, Jones DTW, Pfaff E, Jacob K, Sturm D, Fontebasso AM, Quang D-AK, Tönjes M, Hovestadt V, Albrecht S, Kool M, Nantel A, Konermann C, Lindroth A, Jäger N, Rausch T, Ryzhova M, Korbel JO, Hielscher T, Hauser P, Garami M, Klekner A, Bognar L, Ebinger M, Schuhmann MU, Scheurlen W, Pekrun A, Frühwald MC, Roggendorf W, Kramm C, Dürken M, Atkinson J, Lepage P, Montpetit A, Zakrzewska M, Zakrzewski K, Liberski PP, Dong Z, Siegel P, Kulozik AE, Zapatka M, Guha A, Malkin D, Felsberg J, Reifenberger G, von Deimling A, Ichimura K, Collins VP, Witt H, Milde T, Witt O, Zhang C, Castelo-Branco P, Lichter P, Faury D, Tabori U, Plass C, Majewski J, Pfister SM, Jabado N (2012). Driver mutations in histone H3.3 and chromatin remodelling genes in paediatric glioblastoma. Nature.

[bib77] Sethi R, Allen J, Donahue B, Karajannis M, Gardner S, Wisoff J, Kunnakkat S, Mathew J, Zagzag D, Newman K, Narayana A (2011). Prospective neuraxis MRI surveillance reveals a high risk of leptomeningeal dissemination in diffuse intrinsic pontine glioma. Journal of Neuro-Oncology.

[bib78] Stafford JM, Lee C-H, Voigt P, Descostes N, Saldaña-Meyer R, Yu J-R, Leroy G, Oksuz O, Chapman JR, Suarez F, Modrek AS, Bayin NS, Placantonakis DG, Karajannis MA, Snuderl M, Ueberheide B, Reinberg D (2018). Multiple modes of PRC2 inhibition elicit global chromatin alterations in H3K27M pediatric glioma. Science Advances.

[bib79] Sturm D, Witt H, Hovestadt V, Khuong-Quang D-A, Jones DTW, Konermann C, Pfaff E, Tönjes M, Sill M, Bender S, Kool M, Zapatka M, Becker N, Zucknick M, Hielscher T, Liu X-Y, Fontebasso AM, Ryzhova M, Albrecht S, Jacob K, Wolter M, Ebinger M, Schuhmann MU, van Meter T, Frühwald MC, Hauch H, Pekrun A, Radlwimmer B, Niehues T, von Komorowski G, Dürken M, Kulozik AE, Madden J, Donson A, Foreman NK, Drissi R, Fouladi M, Scheurlen W, von Deimling A, Monoranu C, Roggendorf W, Herold-Mende C, Unterberg A, Kramm CM, Felsberg J, Hartmann C, Wiestler B, Wick W, Milde T, Witt O, Lindroth AM, Schwartzentruber J, Faury D, Fleming A, Zakrzewska M, Liberski PP, Zakrzewski K, Hauser P, Garami M, Klekner A, Bognar L, Morrissy S, Cavalli F, Taylor MD, van Sluis P, Koster J, Versteeg R, Volckmann R, Mikkelsen T, Aldape K, Reifenberger G, Collins VP, Majewski J, Korshunov A, Lichter P, Plass C, Jabado N, Pfister SM (2012). Hotspot mutations in H3F3A and IDH1 define distinct epigenetic and biological subgroups of glioblastoma. Cancer Cell.

[bib80] Sulman EP, Eisenstat DD (2021). World Cancer Day 2021 - perspectives in pediatric and adult neuro-oncology. Frontiers in Oncology.

[bib81] Sun Y, Yan K, Wang Y, Xu C, Wang D, Zhou W, Guo S, Han Y, Tang L, Shao Y, Shan S, Zhang QC, Tang Y, Zhang L, Xi Q (2022). Context-dependent tumor-suppressive BMP signaling in diffuse intrinsic pontine glioma regulates stemness through epigenetic regulation of CXXC5. Nature Cancer.

[bib82] Tajer B, Dutko JA, Little SC, Mullins MC (2021). BMP heterodimers signal via distinct type I receptor class functions. PNAS.

[bib83] Taylor KR, Mackay A, Truffaux N, Butterfield Y, Morozova O, Philippe C, Castel D, Grasso CS, Vinci M, Carvalho D, Carcaboso AM, de Torres C, Cruz O, Mora J, Entz-Werle N, Ingram WJ, Monje M, Hargrave D, Bullock AN, Puget S, Yip S, Jones C, Grill J (2014). Recurrent activating ACVR1 mutations in diffuse intrinsic pontine glioma. Nature Genetics.

[bib84] Tseng WP, Yang SN, Lai CH, Tang CH (2010). Hypoxia induces BMP-2 expression via ILK, Akt, mTOR, and HIF-1 pathways in osteoblasts. Journal of Cellular Physiology.

[bib85] Villanueva RAM, Chen ZJ (2019). Ggplot2: elegant graphics for data analysis.

[bib86] Vitanza NA, Monje M (2019). Diffuse intrinsic pontine glioma: from diagnosis to next-generation clinical trials. Current Treatment Options in Neurology.

[bib87] Wilson PA, Hemmati-Brivanlou A (1995). Induction of epidermis and inhibition of neural fate by Bmp-4. Nature.

[bib88] Wilson SI, Graziano E, Harland R, Jessell TM, Edlund T (2000). An early requirement for FGF signalling in the acquisition of neural cell fate in the chick embryo. Current Biology.

[bib89] Wu G, Broniscer A, McEachron TA, Lu C, Paugh BS, Becksfort J, Qu C, Ding L, Huether R, Parker M, Zhang J, Gajjar A, Dyer MA, Mullighan CG, Gilbertson RJ, Mardis ER, Wilson RK, Downing JR, Ellison DW, Zhang J, Baker SJ (2012). Somatic histone H3 alterations in pediatric diffuse intrinsic pontine gliomas and non-brainstem glioblastomas. Nature Genetics.

[bib90] Wu G, Diaz AK, Paugh BS, Rankin SL, Ju B, Li Y, Zhu X, Qu C, Chen X, Zhang J, Easton J, Edmonson M, Ma X, Lu C, Nagahawatte P, Hedlund E, Rusch M, Pounds S, Lin T, Onar-Thomas A, Huether R, Kriwacki R, Parker M, Gupta P, Becksfort J, Wei L, Mulder HL, Boggs K, Vadodaria B, Yergeau D, Russell JC, Ochoa K, Fulton RS, Fulton LL, Jones C, Boop FA, Broniscer A, Wetmore C, Gajjar A, Ding L, Mardis ER, Wilson RK, Taylor MR, Downing JR, Ellison DW, Zhang J, Baker SJ (2014). The genomic landscape of diffuse intrinsic pontine glioma and pediatric non-brainstem high-grade glioma. Nature Genetics.

[bib91] Wu J-C, Huang C-C, Wang P-W, Chen T-Y, Hsu W-M, Chuang J-H, Chuang H-C (2023). ONC201 suppresses neuroblastoma growth by interrupting mitochondrial function and reactivating nuclear ATRX expression while decreasing MYCN. International Journal of Molecular Sciences.

[bib92] Xie XP, Laks DR, Sun D, Ganbold M, Wang Z, Pedraza AM, Bale T, Tabar V, Brennan C, Zhou X, Parada LF (2022). Quiescent human glioblastoma cancer stem cells drive tumor initiation, expansion, and recurrence following chemotherapy. Developmental Cell.

