# Supplementary figures and images for: BMP2 and BMP7 cooperate with H3.3K27M to promote quiescence and invasiveness in pediatric diffuse midline gliomas
Source: eLife. 2024 Oct 7;12:RP91313. doi: 10.7554/eLife.91313 (PMC11458179; doi:10.7554/eLife.91313)

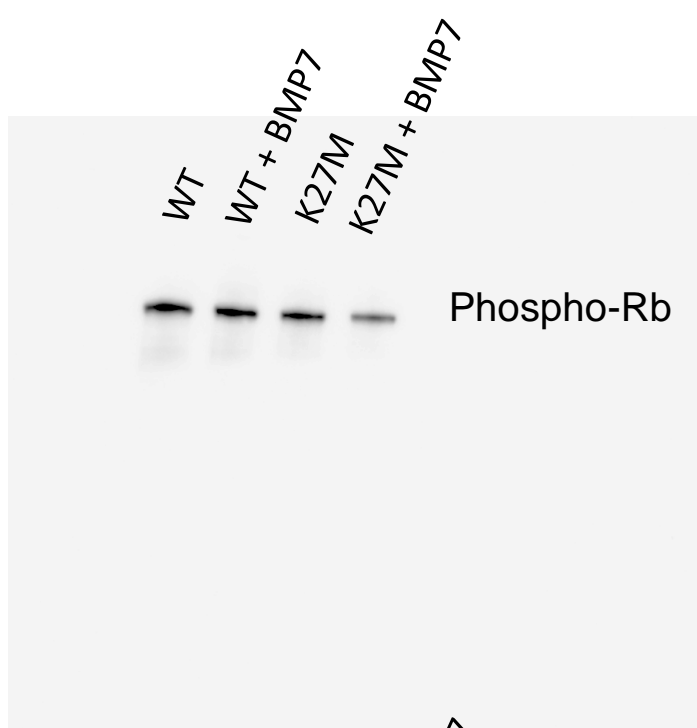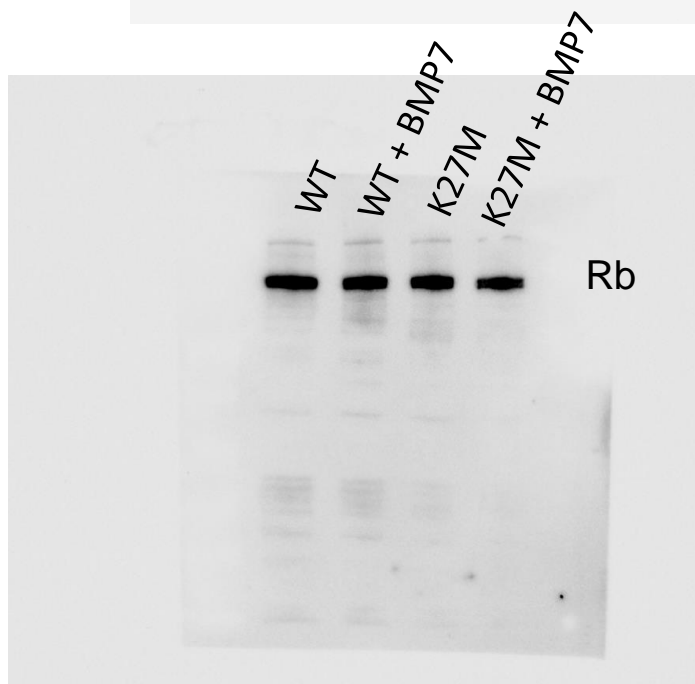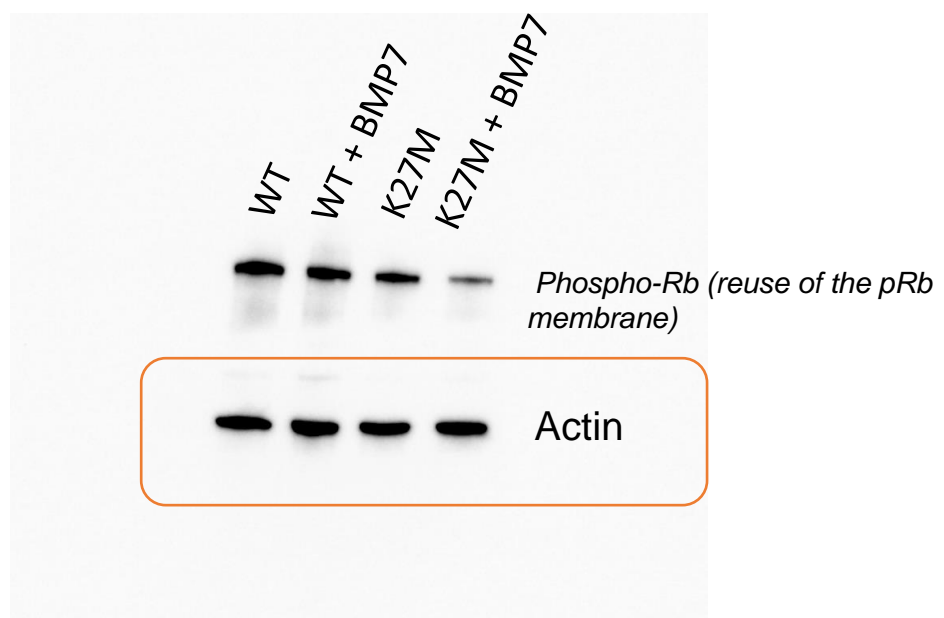

Supplement: Figure 2—source data 1. [file elife-91313-fig2-data1.zip › Figure 2 - source data 1. Uncropped and labelled gels for Figure 2H/Figure 2 - source data 1.pdf]

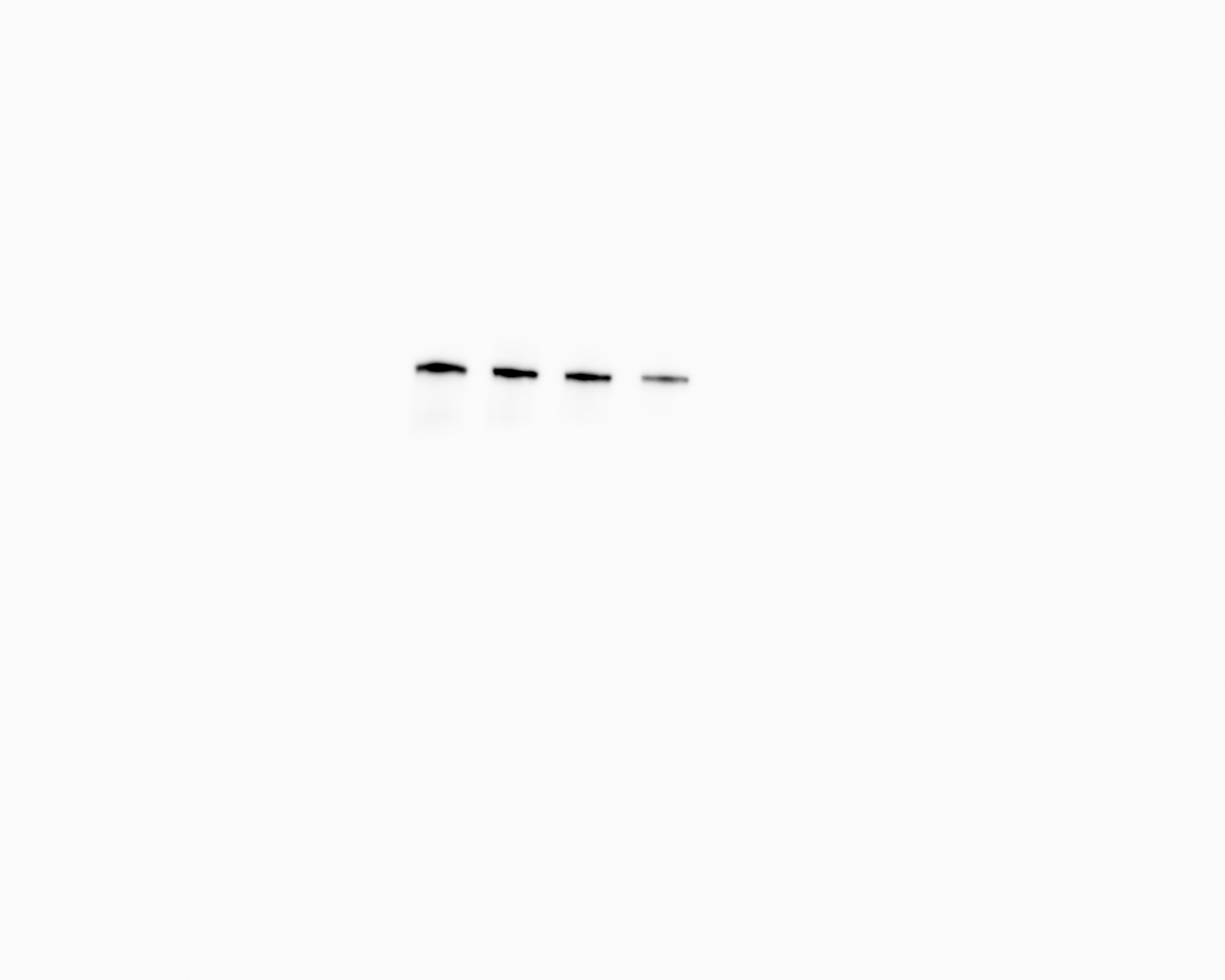

Supplement: Figure 2—source data 2. [file elife-91313-fig2-data2.zip › Figure 2 - source data 2. Raw unedited gels for Figure 2H/Figure 2 - source data 1.tif]

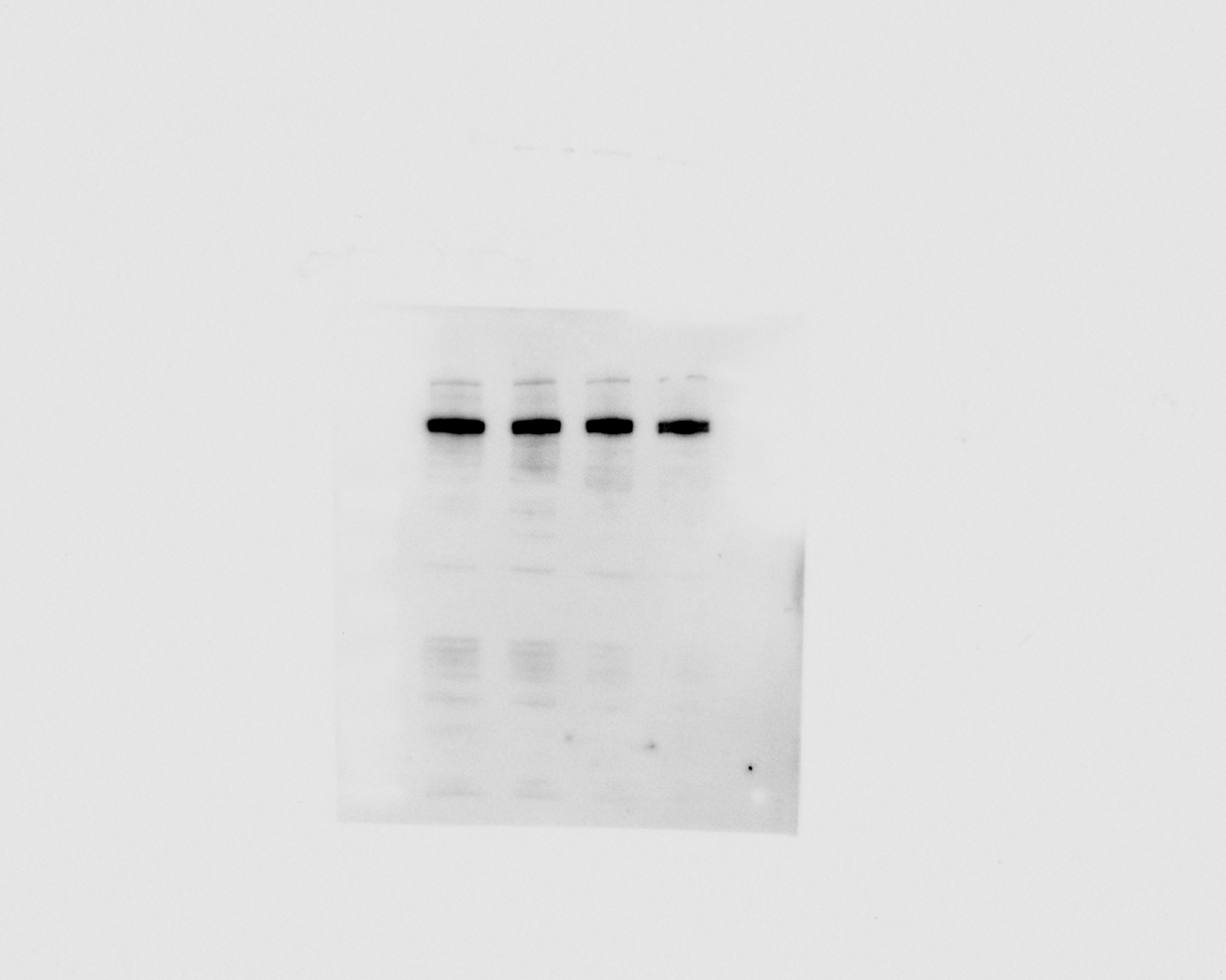

Supplement: Figure 2—source data 2. [file elife-91313-fig2-data2.zip › Figure 2 - source data 2. Raw unedited gels for Figure 2H/Figure 2 - source data 2.tif]

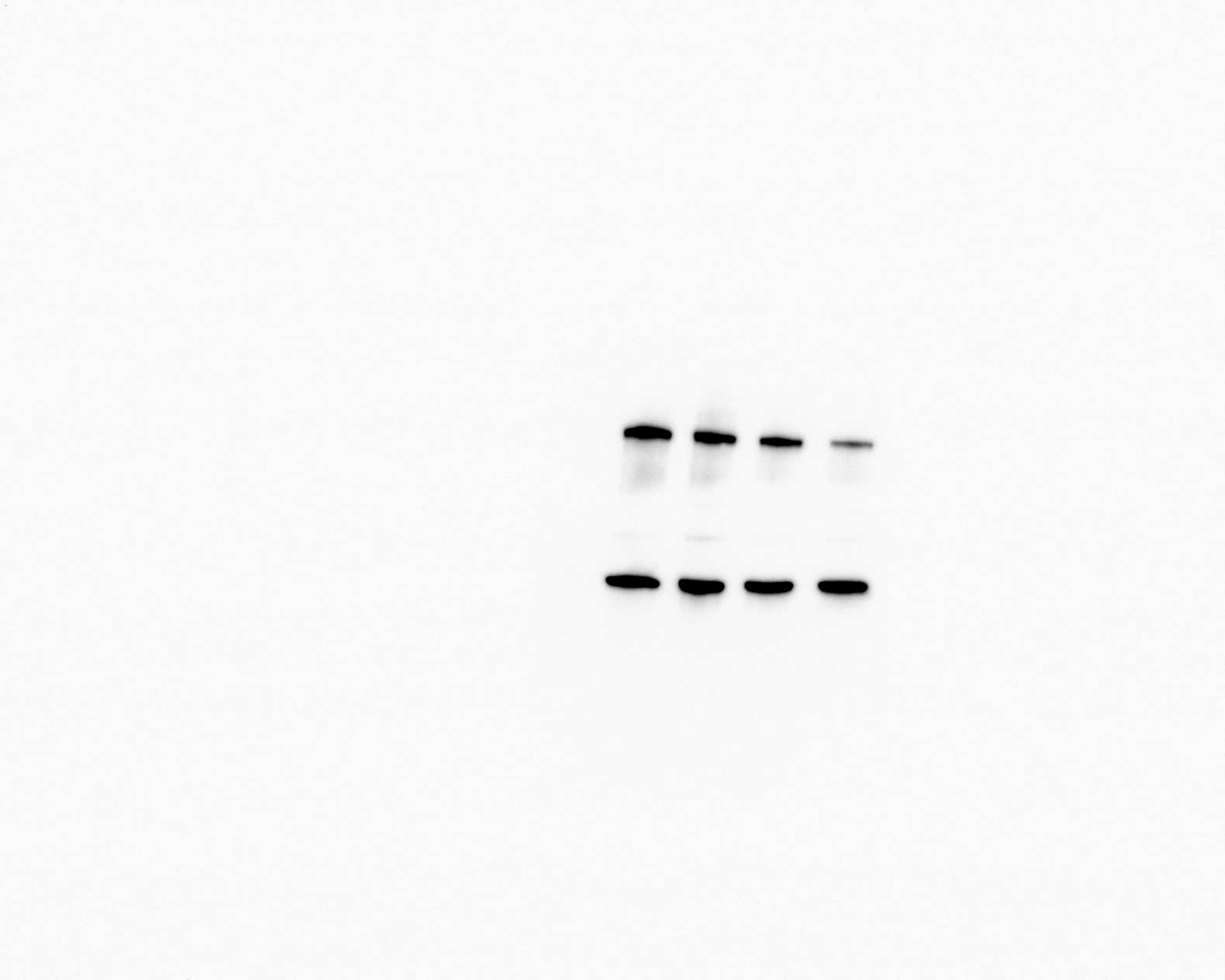

Supplement: Figure 2—source data 2. [file elife-91313-fig2-data2.zip › Figure 2 - source data 2. Raw unedited gels for Figure 2H/Figure 2 - source data 3.tif]

pSMAD1/5/8

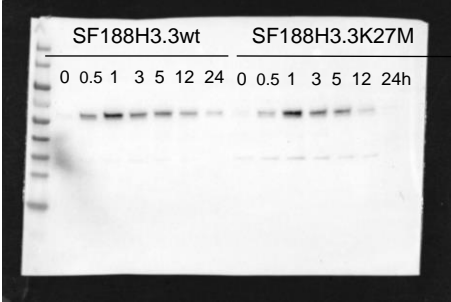

SMAD1

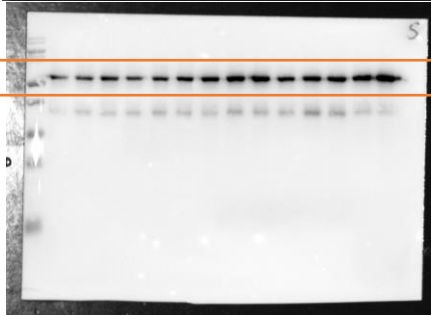

Actin

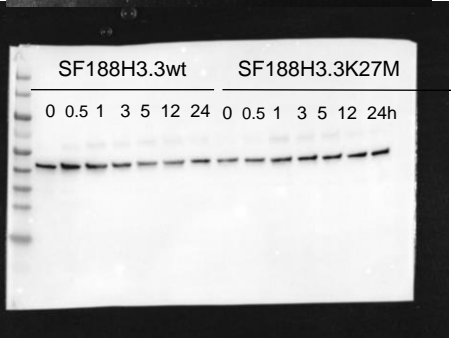

pSMAD1/5/8

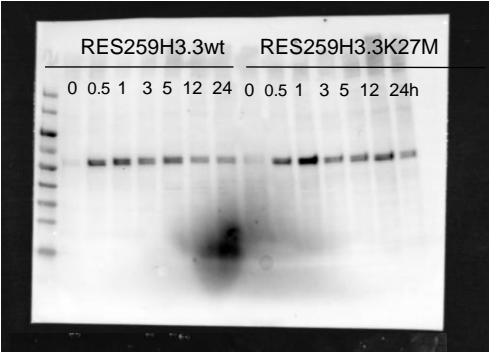

SMAD1

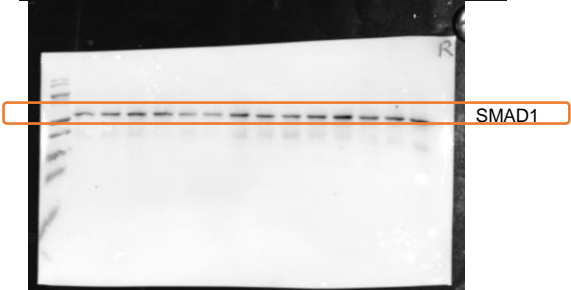

pSMAD1/5/8 band (reuse of the pSMAD membrane)

Actin

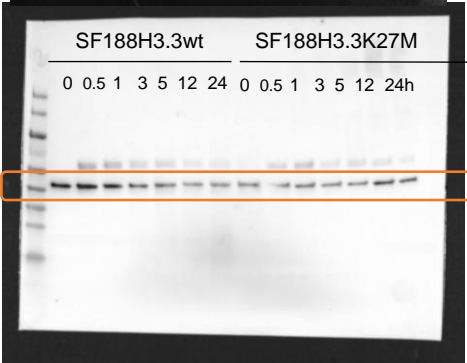

Supplement: Figure 2—figure supplement 1—source data 1. [file elife-91313-fig2-figsupp1-data1.zip › Figure 2 - figure supplement 1 - source data 1. Uncropped and labelled gels for Figure S2D/Figure 2 - figure supplement 1 - source data 1.pdf]

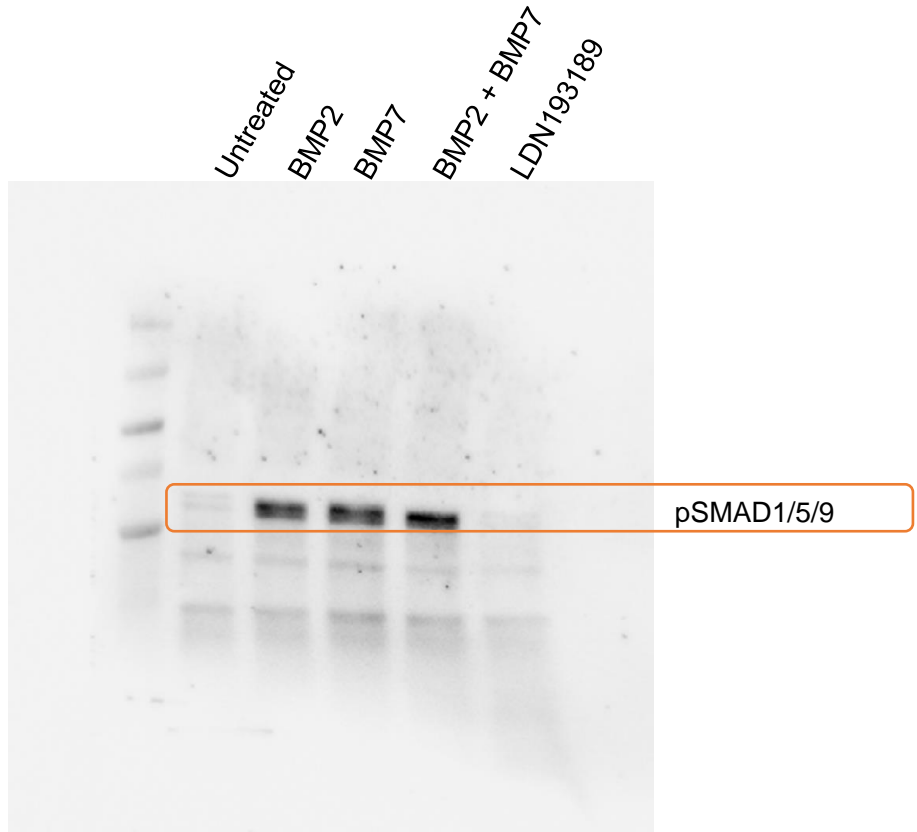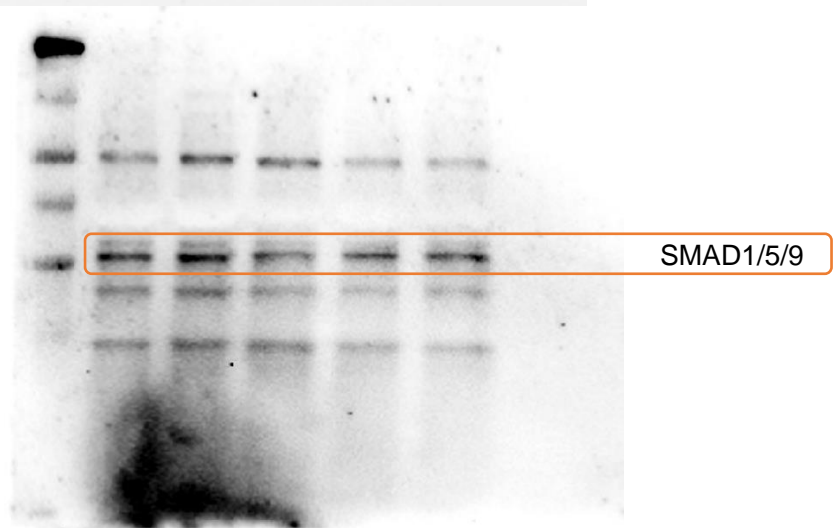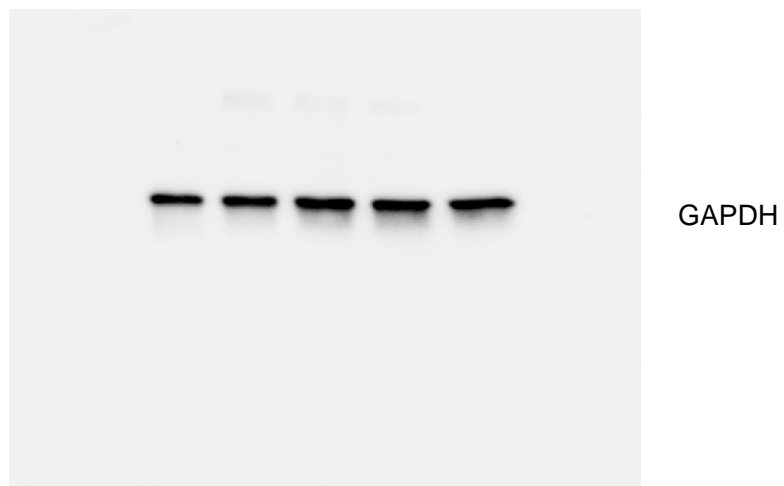

Supplement: Figure 3—figure supplement 1—source data 1. [file elife-91313-fig3-figsupp1-data1.zip › Figure 3 - figure supplement 1 - source data 1. Uncropped and labelled gels for Figure S3B/Figure 3 - figure supplement 1 - source data 1.pdf]

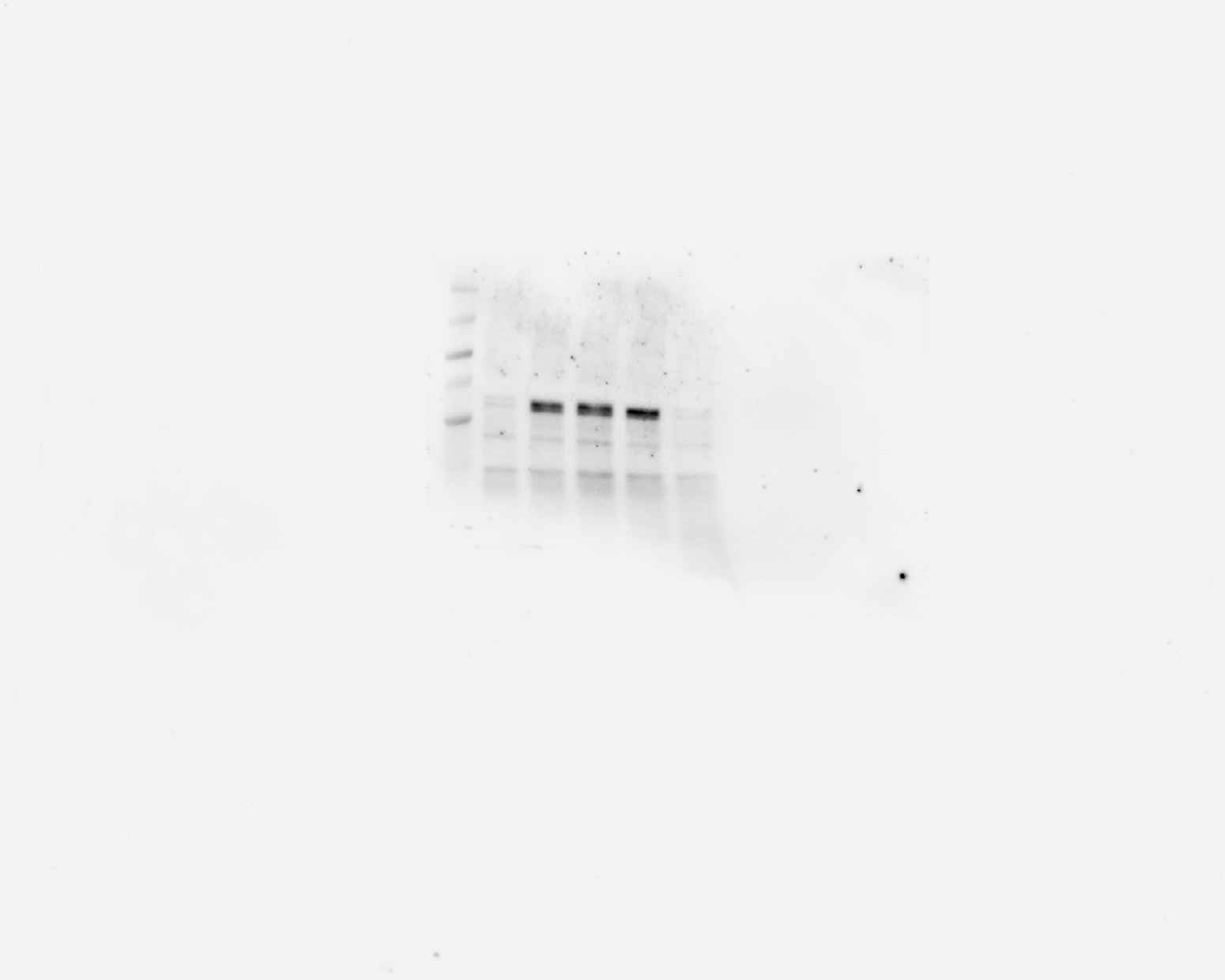

Supplement: Figure 3—figure supplement 1—source data 2. [file elife-91313-fig3-figsupp1-data2.zip › Figure 3 - figure supplement 1 - source data 2. Raw unedited gels for Figure S3B/Figure 3 - supplement 1 - source data 1.tif]

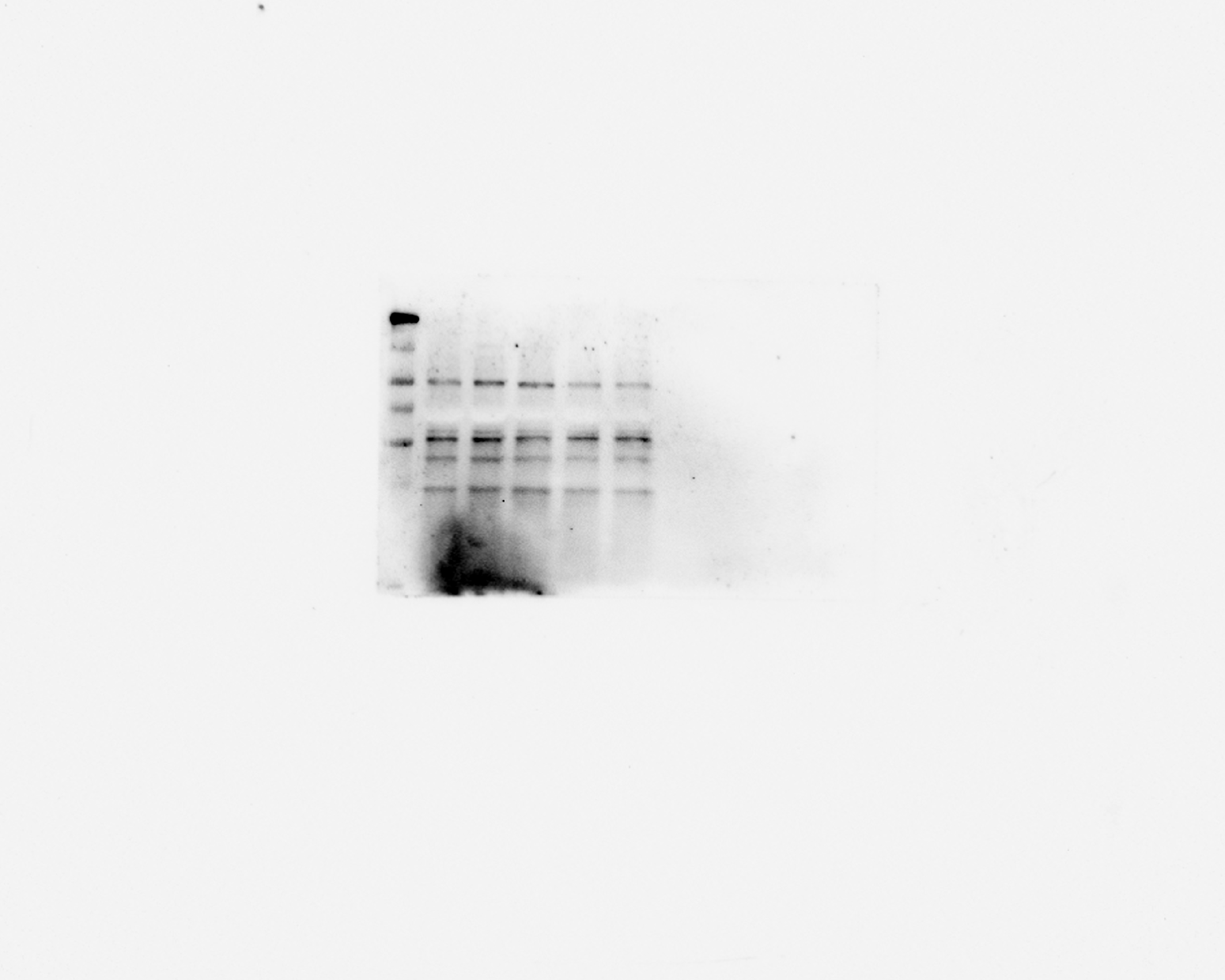

Supplement: Figure 3—figure supplement 1—source data 2. [file elife-91313-fig3-figsupp1-data2.zip › Figure 3 - figure supplement 1 - source data 2. Raw unedited gels for Figure S3B/Figure 3 - supplement 1 - source data 2.tif]

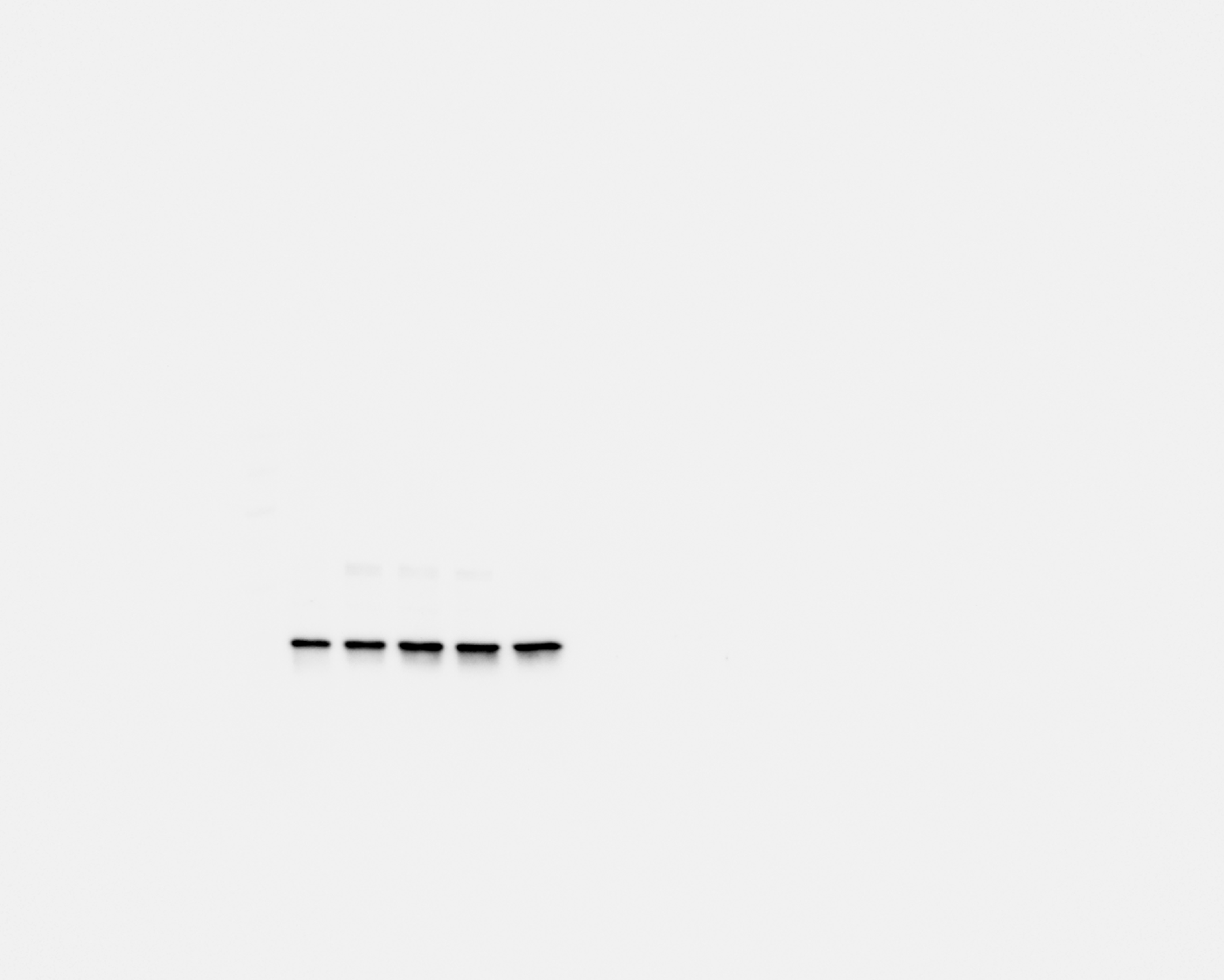

Supplement: Figure 3—figure supplement 1—source data 2. [file elife-91313-fig3-figsupp1-data2.zip › Figure 3 - figure supplement 1 - source data 2. Raw unedited gels for Figure S3B/Figure 3 - supplement 1 - source data 3.tif]
